# Aging and CMV Infection Affect Pre-existing SARS-CoV-2-Reactive CD8^+^ T Cells in Unexposed Individuals

**DOI:** 10.3389/fragi.2021.719342

**Published:** 2021-08-10

**Authors:** Norihide Jo, Rui Zhang, Hideki Ueno, Takuya Yamamoto, Daniela Weiskopf, Miki Nagao, Shinya Yamanaka, Yoko Hamazaki

**Affiliations:** ^1^ Department of Life Science Frontiers, Center for iPS Cell Research and Application (CiRA), Kyoto University, Kyoto, Japan; ^2^ Alliance Laboratory for Advanced Medical Research, Graduate School of Medicine, Kyoto University, Kyoto, Japan; ^3^ Department of Immunology, Graduate School of Medicine, Kyoto University, Kyoto, Japan; ^4^ Institute for the Advanced Study of Human Biology (WPI-ASHBi), Kyoto University, Kyoto, Japan; ^5^ Center for Infectious Disease and Vaccine Research, La Jolla Institute for Immunology, La Jolla, CA, United States; ^6^ Department of Clinical Laboratory Medicine, Graduate School of Medicine, Kyoto University, Kyoto, Japan; ^7^ Gladstone Institute of Cardiovascular Disease, San Francisco, CA, United States; ^8^ Laboratory of Immunobiology, Graduate School of Medicine, Kyoto University, Kyoto, Japan

**Keywords:** SARS-CoV-2, COVID-19, T-cell immunity, cross-reactive T cells, senescent T cells, cytomegalovirus, T cell aging

## Abstract

Age is a major risk factor for COVID-19 severity, and T cells play a central role in anti-SARS-CoV-2 immunity. Because SARS-CoV-2-cross-reactive T cells have been detected in unexposed individuals, we investigated the age-related differences in pre-existing SARS-CoV-2-reactive T cells. SARS-CoV-2-reactive CD4^+^ T cells from young and elderly individuals were mainly detected in the central memory fraction and exhibited similar functionalities and numbers. Naïve-phenotype SARS-CoV-2-reactive CD8^+^ T cell populations decreased markedly in the elderly, while those with terminally differentiated and senescent phenotypes increased. Furthermore, senescent SARS-CoV-2-reactive CD8^+^ T cell populations were higher in cytomegalovirus seropositive young individuals compared to seronegative ones. Our findings suggest that age-related differences in pre-existing SARS-CoV-2-reactive CD8^+^ T cells may explain the poor outcomes in elderly patients and that cytomegalovirus infection is a potential factor affecting CD8^+^ T cell immunity against SARS-CoV-2. Thus, this study provides insights for developing effective therapeutic and vaccination strategies for the elderly.

## Introduction

Aging is the most significant risk factor for severe outcomes and high mortality in coronavirus disease (COVID-19) patients (Banerjee et al., 2020; Onder et al., 2020; Richardson et al., 2020; Wu et al., 2020). Higher susceptibility to disease and death in aging populations is a major challenge for developing vaccines and immunotherapeutic agents. With age, the integrity of the immune system gradually deteriorates and is reflected in diminished acquired immunity and increased inflammatory traits (Goronzy et al., 2013). T cells play a central role in adaptive immune responses upon viral infections; however, their number, composition, and functionality change remarkably with age (Kumar et al., 2018; Nikolich-Zugich, 2018; Goronzy and Weyand, 2019; Mittelbrunn and Kroemer, 2021; Zhang et al., 2021). It has been suggested that the coordination of antigen-specific adaptive immune responses by severe acute respiratory syndrome coronavirus 2 (SARS-CoV-2)-specific CD4^+^ T cells and CD8^+^ T cells limits the severity of COVID-19 but that this coordination is disrupted in elderly individuals (≥65 years of age) (Rydyznski Moderbacher et al., 2020). Specifically, correlations among low frequencies of naïve CD4^+^ and CD8^+^ T cells, age, and COVID-19 disease severity have been observed (Rydyznski Moderbacher et al., 2020; Zhang et al., 2020); however, whether these observations are a cause or consequence of severe COVID-19 that causes a drastic change in T cell phenotypes, including lymphopenia (Chen and John Wherry, 2020; Huang et al., 2020), is not well understood.

The qualitative and quantitative changes in T cell phenotypes with age are partly attributable to shrinkage of the thymus, a primary lymphoid organ responsible for naïve T cell generation. The phenomenon of thymic involution is already evident during adolescence, and the organ is gradually replaced with adipose tissues by the middle and later ages (Lynch et al., 2009; Chaudhry et al., 2016). Accordingly, the rate of thymic T-cell output (around 2 × 10^6^ cells per day at peak) declines over time, with an estimated half-life of approximately 16 years in humans (Murray et al., 2003). Although the peripheral naïve T cell number is sustained by the homeostatic proliferation of peripheral T cells (Murray et al., 2003; den Braber et al., 2012) to some extent, the proportion as well as the number of naïve T cells, especially CD8^+^ naïve T cells, decreases significantly with age (Wertheimer et al., 2014). Since naïve T cells ensure reactivity against unexposed antigens, the decrease in naïve T cells in elderly individuals may theoretically be disadvantageous upon exposure to SARS-CoV-2.

T-cell response to novel pathogens is also elicited via cross-recognition by memory phenotype (MP) T cells generated by exposure to other antigens (Sewell, 2012; Su et al., 2013). Such cross-reactive memory T cells could be frequently generated by infection and/or vaccination with the same virus species. In the case of influenza, the presence of pre-existing cross-reactive T cells against conserved influenza viral proteins correlates with lower severity of H1N1 flu, suggesting their protective effects (Greenbaum et al., 2009; Wilkinson et al., 2012; Sridhar et al., 2013). Although their impact on susceptibility to and/or severity of COVID-19 is still controversial (Lipsitch et al., 2020), SARS-CoV-2-reactive MP (cross-reactive) T cells have been detected in unexposed individuals (Bacher et al., 2020; Braun et al., 2020; Grifoni et al., 2020; Le Bert et al., 2020; Mateus et al., 2020; Sekine et al., 2020; Thieme et al., 2020; Weiskopf et al., 2020). Considering that T cell composition dramatically changes throughout life (Kumar et al., 2018), the number or functionalities of pre-existing SARS-CoV-2-reactive T cells with the MP as well as naïve phenotype (NP) could change with age, thus explaining the age-related differences in COVID-19 severity.

In addition to the proportional changes in NP and MP T cells, recent studies have revealed that T cell populations with highly differentiated phenotypes and/or cellular senescence characteristics accumulate with age; senescent T cells show an increased expression of DNA damage markers [e.g., phosphorylated histone H2AX (γH2AX)], shortened telomeres, and low telomerase activity (Akbar et al., 2016). We previously reported that T-cell senescence was induced by prolonged homeostatic proliferation to compensate for the decrease in thymic T cell production in a mouse model (Sato et al., 2017; Kato et al., 2018; Minato et al., 2020). CD57 expression defines replicative senescence in human T cells (Brenchley et al., 2003), and patients thymectomized during early childhood show an early accumulation of CD57^+^ senescent T cells during their lifetime (Sauce et al., 2009). Furthermore, latent virus infections, such as cytomegalovirus (CMV) infection, induce the accumulation of CD57^+^CD28^−^ senescent-like T cells in humans (Henson et al., 2012; Klenerman and Oxenius, 2016). Importantly, these cells retain efficient cytotoxicity and produce high amounts of cytokines but do not proliferate well after antigen challenge and exhibit biased T cell receptor (TCR) repertoires, providing a mechanism for immunosenescence.

This study investigated the mechanism underlying the differences in disease severity upon SARS-CoV-2 infection between young and elderly individuals. To this end, we examined the numbers and frequencies, as well as phenotypes, of SARS-CoV-2-reactive T cells from uninfected individuals in a Japanese cohort.

## Methods and Materials

### Study Design

Donors were required to be 20 years of age or older. Blood samples were collected at Kyoto University Hospital, and HIV, HTLV-1, HBV, and HCV negative samples were used. All donors were recruited on the condition that they were healthy and did not have any serious medical conditions nor history including cancer, gastrointestinal, liver, kidney, cardiovascular, hematologic or endocrine disease. Samples were de-identified with an anonymous code assigned to each sample. Their characteristics, including age, gender, complete blood count and serology, are summarized in Table 1. Samples were obtained between July and September 2020 during the COVID-19 pandemic. Blood samples from convalescents were obtained 6 months after confirmation of SARS-CoV-2 negativity by PCR tests.

**TABLE 1:** Participant characteristics.

	Young (*n* = 30)	Elderly (*n* = 26)	*p* value*
Age, y-median (IQR)	22 (2)	72 (5.5)	<0.001^a^
Gender	—	—	n.s.^b^
Male %(*n*)	50 (15)	69.2 (18)	—
Female %(*n*)	50 (15)	30.8 (8)	—
Complete blood count	—	—	—
WBC × 10^9^/L -medain (IQR)	5.64 (1.96)	5.56 (2.33)	n.s.^a^
Monocytes × 10^9^/L -medain (IQR)	0.38 (0.16)	0.41 (0.14)	n.s.^a^
Lymphocytes × 10^9^/L -medain (IQR)	1.92 (0.58)	1.55 (0.67)	0.0105^a^
CD3^+^ T cells × 10^9^/L -medain (IQR)	1.38 (0.39)	1.07 (0.37)	0.0023^a^
CD4^+^ T cells × 10^9^/L -medain (IQR)	0.71 (0.25)	0.71 (0.35)	n.s.^a^
CD8^+^ T cells × 10^9^/L -medain (IQR)	0.46 (0.22)	0.24 (0.14)	<0.001^a^
Serology	—	—	—
CMV IgG positivity % (*n*)	46.7 (14)	100 (26)	<0.001^b^
CMV IgG titers in carriers AU/mL -medain (IQR)	106.8 (144.15)	213 (54.25)	0.0039^a^
SARS-COV-2 Ig Positivity % (*n*)	0 (0)	0 (0)	n.s^b^

a Mann-Whiteney test.

b Fisher’s exact test.

### PBMC Isolation, Cryopreservation, and Thawing

Whole blood was drawn into a BD Vacutainer CPT^TM^ Cell Preparation Tube with sodium heparin and processed within 2 h to isolate peripheral blood mononuclear cells (PBMCs) according to the manufacturer’s instructions. Isolated PBMCs were cryopreserved in CELLBANKER (ZENOGEN PHARMA) 1 at 8 × 10⁶ cells/ml and stored in a −150°C ultra-low temperature freezer until used in the assays. Cryopreserved PBMCs were thawed in pre-warmed X-VIVO15 (LONZA) without serum. After centrifugation, the cells were washed once and directly used for the assay as described below.

### Complete Blood Counts

Whole blood was collected in an EDTA-2Na tube. The analysis was performed using the Automated Hematology Analyzer XN-9000 (Sysmex Corporation) at the Department of Clinical Laboratory, Kyoto University Hospital.

### Serology

Whole blood was collected in a blood collection vessel, Venoject VP-P075K (Terumo), for serum isolation. Serum separator tubes were centrifuged for 4 min at 1,100 rcf at 4°C. The serum was then removed from the upper portion of the tube, aliquoted, and stored at −80°C. Anti-SARS-CoV-2 IgM/IgG levels in the serum were measured using Elecsys Anti-SARS-CoV-2 with cobas8000 (Roche Diagnostics K.K.) at the Department of Clinical Laboratory, Kyoto University Hospital. Anti-CMV IgG levels were measured using chemiluminescence immunoassays (CLIA) at LSI medience (Tokyo, Japan). The cut-off values for Anti-SARS-CoV-2 IgM/IgG and Anti-CMV IgG were 1.0 cutoff index (COI) and 6.0 (AU/ml), respectively.

### Peptide Pools

PepTivator SARS-CoV-2 peptide pools (Miltenyi Biotech) were diluted in distilled water (DW) and used for the SARS-CoV-2-reactive T cell stimulation. The S peptide pool contained 15-mer peptides that overlapped by 11 amino acids and spanned the immunodominant sequence domains of the spike (S) glycoprotein of SARS-CoV-2. The S1, N and M peptide pools covered the entire sequence of the corresponding proteins. All four peptide pools were equally combined at the final concentration of 0.6 nmol/ml in the culture medium. CMV pp65 peptide pools (Miltenyi Biotech) were used for CMV-reactive T cell stimulation.

### Activation-Induced Marker Assay

PBMCs were cultured in 150 μl X-VIVO15 medium supplemented with 5% human AB serum (Sigma) for 20 h at 37°C in the presence of SARS-CoV-2 or CMV peptide pools (0.6 nmol/ml) and CD40 blocking antibody (Miltenyi Biotech) (0.5 ug/ml) in 96-well U-bottom plates at 2 × 10^6^ PBMCs per well. Stimulation with DW at an equal volume was performed as a negative control. After the stimulation, the cells were stained with Ghost Dye^TM^ Red 710 (TONBO) to discriminate viable from non-viable cells. The cells were then washed and stained with fluorochrome-conjugated surface antibodies at pre-titrated concentrations in the presence of FcR blocking (Miltenyi Biotech) for 20 min at 4°C. Antibodies used in the AIM assay are listed in Supplementary Table 1. Stained cells were fixed and permeabilized for 20 min at room temperature using the eBioscience Foxp3/Transcription Factor Staining Buffer Set. The cells were then stained with anti-IRF4 antibody (1:500 dilution) for 30 min at room temperature. After the final wash, the cells were resuspended in 200 μl PBS with 2% FBS (FACS buffer) for flow cytometry analysis. Supernatants were harvested at 20 h post-stimulation for the multiplex detection of cytokines.

### Intracellular Cytokine Staining (ICS) After Peptide Stimulation Assay

PBMCs were first stained with 2 µM Cell Trace Violet (CTV) (Thermo Fisher Scientific) for 15 min and washed with X-VIVO15 medium. The labeled cells were cultured in 200 μl X-VIVO15 medium supplemented with 2% human AB serum for 6 days at 37°C in the presence of SARS-CoV-2 peptide pools (0.6 nmol/ml) at 1 × 10^6^ PBMCs per well or Dynabeads Human T-Activator CD3/CD28 (Thermo Fisher Scientific) at 1 × 10^5^ PBMCs per well in 96-well U-bottom plates. A stimulation with an equal volume of DW was performed as the negative control. The medium change was conducted every three days. Five hours before ICS, the medium was replaced to fresh medium with the respective peptide pool and brefeldin A (BioLegend) (1:1,000 dilution). After stimulation, the cells were stained with Ghost Dye^TM^ Red 710 to discriminate viable from non-viable cells. Cells were then washed and stained with fluorochrome-conjugated surface antibodies at pre-titrated concentrations in the presence of FcR blocking for 20 min at 4°C. The antibodies used in the ICS assay are listed in Supplementary Table 2. Stained cells were then fixed and permeabilized using IC fixation buffer and Permeabilization buffer for 20 min at room temperature. The cells were then stained for intracellular IL-2, IL-17A, IFNγ, and TNFα (1:25 dilution for each) for 30 min at room temperature. After the final wash, the cells were resuspended in 200 μl FACS buffer for flow cytometry analysis.

### Flow Cytometry and FCS Data Analysis

All the samples in the AIM and ICS assays were acquired using a BD FACSAria™ II Cell Sorter (BD Biosciences). FCS 3.0 data files were exported and analyzed with FlowJo software version 10.6.1. Detailed gating strategies for individual markers are described in Supplementary Figures 2, 4, and 5.

### Cytokine Bead Assays

Supernatants were collected from the culture plate after 20 h of AIM assays and after 3 or 6 days of peptide pool stimulation. They were then stored in 96-well plates at −80 °C until use. Cytokines in the cell culture supernatants were quantified using a human Th cytokine panel (12-plex) kit (BioLegend) according to the manufacturer’s instruction. Briefly, the supernatants were mixed with beads coated with capture antibodies specific for IL-5, IL-13, IL-2, IL-6, IL-9, IL-10, IFNγ, TNFα, IL-17A, IL-17F, IL-4 and IL-22 and incubated on a 96-well filter plate for 2 h. The beads were washed and incubated with biotin-labeled detection antibodies for 1 h, followed by a final incubation with streptavidin-PE. The beads were analyzed by flow cytometry using a BD LSR flow cytometer (BD Biosciences). Analysis was performed using the LEGENDplex analysis software v8.0, which distinguishes the 12 different analytes based on the bead size and internal dye.

### opt-SNE

Dimensionality reduction of multi-color flow cytometry data obtained from the AIM assays was performed using OMIQ software. FCS 3.0 data from all donors were imported. Up to 117 cells of AIM^+^CD4^+^ and 37 cells of AIM^+^CD8^+^ T cells from each donor were subsampled and merged for the analysis. These subsampling counts were derived from the median in the corresponding subsets. The markers we applied to the opt-SNE analysis are described in the figure legends. The parameters used were as follows: Max Iterations = 1,000, opt-SNE End = 5,000, Perplexity = 30, Theta = 0.5, Components = 2, Random Seed = 6,925, Verbosity = 25.

### Graphic Representation

Subset definitions and gating strategies are outlined in the text and/or figure legends. The absolute numbers of a defined subset (e.g., NP, CM, EM, TEMRA, and AIM^+^ cells) for CD4^+^ and CD8^+^ T cells were obtained by individually multiplying the number of CD4^+^ or CD8^+^ T cells by the percentage of the corresponding subsets. The percentage of AIM^+^ T cells was calculated by subtracting the percentage of AIM^+^ cells after DW treatment from that after peptide stimulation. To calculate the percentages of each subset, samples that a percentage of AIM^+^ cells in CD4 or CD8 T cells of at least 0.001% are indicated in the graphs, since a very low frequency of AIM^+^ cells influences the results. The stimulation index (SI) was calculated by dividing the percentage of AIM^+^ cells after SARS-CoV-2 peptide pool stimulation with that after DW treatment. If the percentage of AIM^+^ cells after DW stimulation equaled 0, the minimum value across each cohort was used instead.

### Statistical Analysis

All statistical analyses in this study were performed using GraphPad Prism 9.0. The statistical details, such as number of subjects, cohorts, statistical test and significance of the experiments, are provided in the respective figure legends. Non-parametric tests were primarily used for the statistical analysis in this study, because most of the sample values had some outliers and did not follow a Gaussian distribution, which was assessed by the Shapiro-Wilk normality test. Two-tailed Mann-Whitney and Wilcoxon tests were applied for unpaired and paired group comparisons, respectively. Correlation analyses were performed using the Spearman rank correlation test.

## Results

### Young and Elderly Unexposed Individuals Harbor Comparable Numbers of SARS-CoV-2-Reactive T Cells

Thirty young (median age, 22 years) and twenty-six elderly (median age, 72 years), healthy, uninfected participants were recruited (Table 1). All donors were anti-SARS-CoV-2 Ig negative (Table 1). PBMCs were isolated and used for further analyses. The numbers of white blood cells and monocytes did not differ between the two groups, but lymphocyte and CD3^+^ T cell numbers were approximately 20% lower in the elderly (Table 1; Supplementary Figure 1). The number of CD4^+^ T cells was comparable between the two groups, while the number of CD8^+^ T cells in the elderly was approximately 50% that of the young (Table 1; Supplementary Figure 1) 1), consistent with previous reports showing a more profound reduction of CD8^+^ T cells with age (Czesnikiewicz-Guzik et al., 2008; Wertheimer et al., 2014).

To identify and quantify SARS-CoV-2-reactive T cells in those samples, we utilized TCR-dependent AIM assays, a commonly used and cytokine-independent system to detect antigen-specific T cells (Dan et al., 2016; Havenar-Daughton et al., 2016; Herati et al., 2017; Reiss et al., 2017; Morou et al., 2019), including SARS-CoV-2-reactive T cells in healthy individuals (Bacher et al., 2020; Braun et al., 2020; Grifoni et al., 2020; Mateus et al., 2020; Weiskopf et al., 2020). T cell responses have been detected mainly against the spike (S), membrane (M), and nucleocapsid (N) proteins of SARS-CoV-2 (Braun et al., 2020; Grifoni et al., 2020; Le Bert et al., 2020; Mateus et al., 2020; Sekine et al., 2020; Thieme et al., 2020; Weiskopf et al., 2020). Therefore, to reduce the required quantity of blood samples from an ethical perspective, we used overlapping peptide mixtures that could stimulate both CD4^+^ and CD8^+^ T cells and cover immunodominant sequences of SARS-CoV-2, including the N-terminal and C-terminal regions of S (amino acid residues 1–692 and 885–1,273, respectively), as well as N and M proteins, for PBMC stimulation. Flow cytometry identified antigen-specific AIM^+^ cells and their phenotypes in stimulated PBMCs. The gating strategy is shown in Supplementary Figure 2A. AIM^+^ cells were defined by the co-expression of CD137 and IRF4, which sensitively marks peptide-specific T cells with low background and reflects TCR signal strength (Wu et al., 2019). Utilizing CMV-pp65 peptide stimulation, AIM^+^(CD137^+^IRF4^+^) CD4^+^ and CD8^+^ T cells were substantially detected in CMV seropositive (CMV^+^) individuals but not in CMV seronegative (CMV^−^) individuals (Supplementary Figure 2B). Similar results were obtained using more common AIMs (CD137 and CD154) (Reiss et al., 2017; Grifoni et al., 2020) in CD4^+^ T cells (Supplementary Figure 2B). Furthermore, based on SARS-COV-2 peptide stimulations, more than approximately 30-fold higher frequencies of AIM^+^ CD4^+^ and CD8^+^ T cells were detected in convalescent patients with COVID-19 compared to unexposed individuals (Supplementary Figure 2B), confirming the accuracy of the detection system and the AIMs.

**FIGURE 1:**
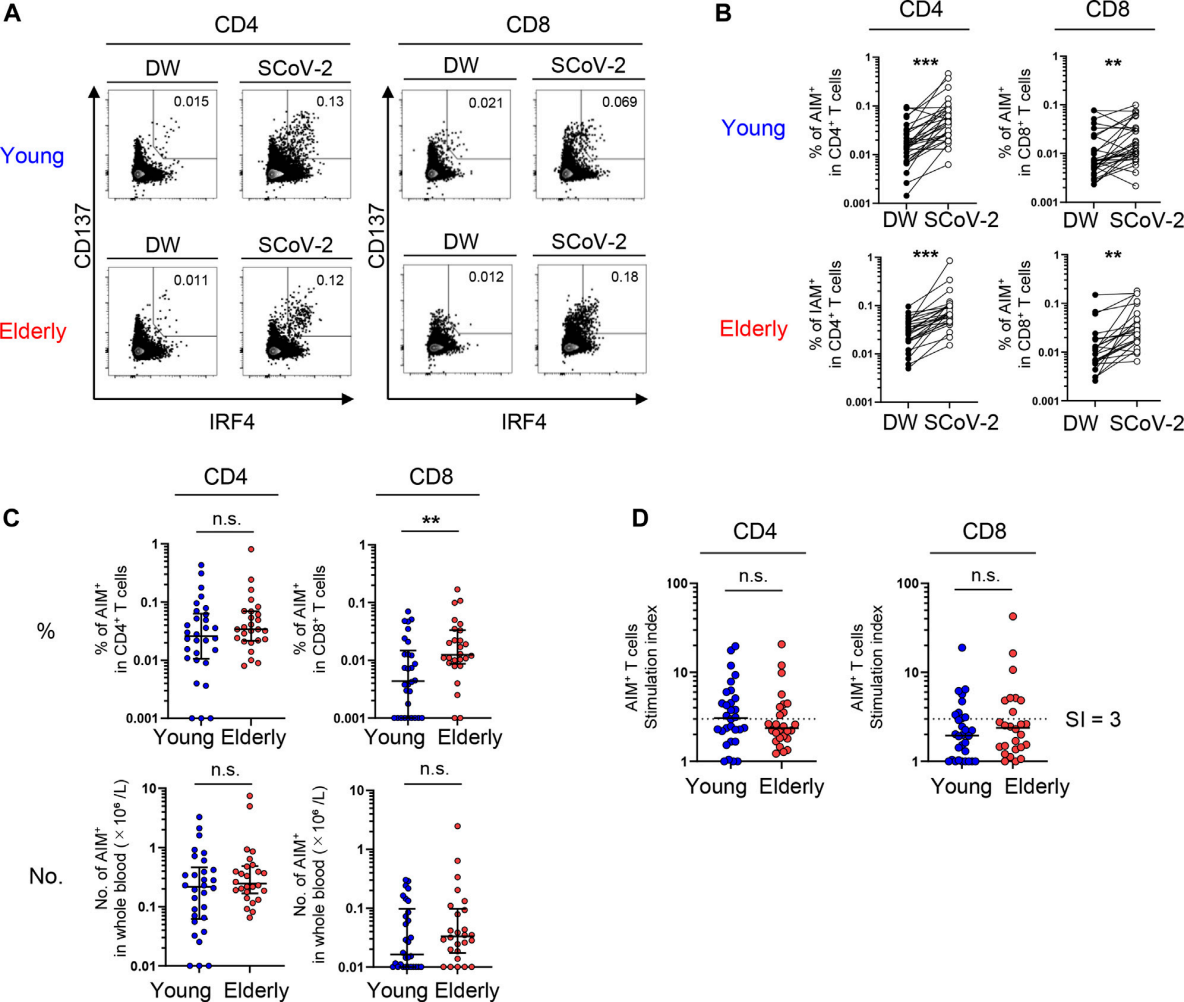
SARS-CoV-2-specific T-cell responses in unexposed young and elderly individuals. PBMCs isolated from 30 young and 26 elderly blood samples were stimulated for 20 h with DW or overlapping peptides containing the SARS-CoV-2 S, N, and M protein sequences. Antigen-reactive T cells were identified by flow cytometry (FCM) according to the gating strategy and staining presented in Supplementary Figure 2A and Supplementary Table 1. **(A)** Representative FCM plots displaying AIMs (CD137 and IRF4) on CD4^+^ and CD8^+^ T cells after stimulation with the negative control (DW) or SARS-CoV-2 peptide pool (SCoV-2). Numbers indicate the population percentages in the gates. **(B)** Percentages of AIM^+^ (CD137^+^IRF4^+^) T cells in CD4^+^ and CD8^+^ T cells between the negative control (DW) and SARS-CoV-2 peptide stimulated samples (SCoV-2). **(C)** Percentages of AIM^+^ (CD137^+^IRF4^+^) T cells in CD4^+^ and CD8^+^ T cells and their numbers (×10⁶/L). Data were background subtracted against the DW and are shown as the median ± interquartile range (IQR). **(D)** Stimulation index (SI) quantification of AIM^+^CD4^+^ or AIM^+^CD8^+^ T cells; the same samples as in Figure 1C were analyzed. The cutoff value was defined as >3, as indicated by the dashed lines. **(B**-**D)** Each dot represents one donor. Pairwise comparisons were performed using Wilcoxon’s test. Statistical comparisons across cohorts were performed using the Mann-Whitney test. **p < 0.01, ***p < 0.001. n.s., not significant.

SARS-CoV-2-specific responses (the percentage of peptide-stimulated AIM^+^ cells subtracted by DW-stimulated cells) were detected in CD4^+^ and CD8^+^ T cells from both young and elderly unexposed individuals (Figures 1A,B). The frequencies and numbers of SARS-CoV-2-reactive CD4^+^ T cells were comparable between the two groups (Figure 1C). Similar results were obtained using another AIM combination (CD137 and CD154) (Supplementary Figure 2C, D), and AIM^+^ frequencies in total CD4^+^ T cells using these two AIM combinations exhibited a positive correlation (Supplementary Figure 2E). On the other hand, the frequency of SARS-CoV-2-reactive cells among total CD8^+^ T cells was significantly higher in the elderly than in the young participants, although the number of SARS-CoV-2-reactive CD8^+^ T cells/L in the blood was not different between the two groups owing to the low CD8^+^ T cell count in the peripheral blood of the elderly (Figure 1C; Table 1; Supplementary Figure 1). Based on the definition of responders using the stimulation index, which was calculated as peptide-specific responses/DW responses (Grifoni et al., 2020; Weiskopf et al., 2020) > 3, we found that CD4^+^ T cells in 50.0% (15/30) and 34.6% (9/26) and CD8^+^ T cells in 30.0% (9/30) and 30.8% (8/23) of young and elderly individuals, respectively, showed a significant level of pre-existing SARS-CoV-2-reactive T cells (Figure 1D). These results indicate that young and elderly unexposed individuals have comparable numbers of SARS-CoV-2-reactive T cells.

### SARS-CoV-2-Reactive CD4^+^ T Cell Phenotypes are Similar, but CD8^+^ T Cell Phenotypes Differ Between the Young and Elderly

**FIGURE 2:**
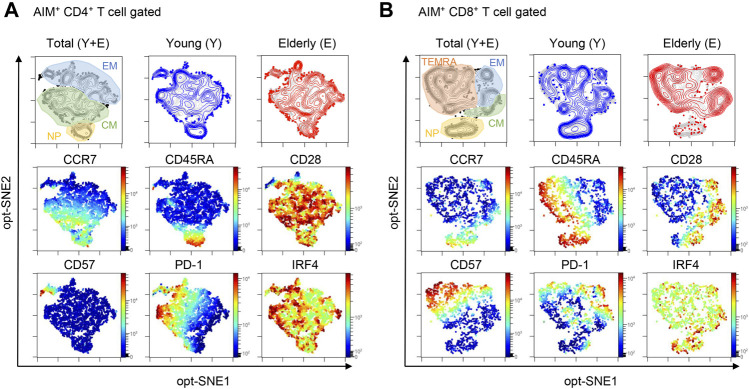
opt-SNE mapping of SARS-CoV-2-reactive T cells from young and elderly individuals. The opt-SNE plots were generated using all the markers described in the **middle** and **bottom** rows (CCR7, CD45RA, CD28, CD57, PD-1, and IRF4). **(Top)** opt-SNE plots showing the clustering of AIM^+^CD4^+^ **(A)** or AIM^+^CD8^+^ **(B)** T cells from young (blue) (*n* = 30) and elderly (red) *(n* = 26) cohorts. Background plots shown in gray represent total AIM^+^CD4^+^ **(A)** and AIM^+^CD8^+^ **(B)** T cells from all 56 samples. Presumable naïve phenotype (NP), central memory (CM), effector memory (EM), and terminally differentiated effector memory cells re-expressing CD45RA (TEMRA) were manually gated based on the expression levels of CD45RA and CCR7. (**Middle** and **Bottom**) opt-SNE plots showing the expression of individual markers. See Supplementary Figure 3.

To obtain a global view of the SARS-CoV-2-reactive T cell phenotype, the data were visualized by optimized t-Distributed Stochastic Neighbor Embedding (opt-SNE) (Belkina et al., 2019). AIM^+^ cells from the young and elderly were overlayed on total AIM^+^ cells from both groups to visualize marker expression patterns. Phenotypically related cells, namely NP, central memory (CM), effector memory (EM), and terminally differentiated effector memory T cells re-expressing CD45RA (TEMRA), were identified using opt-SNE based on CCR7 and CD45RA expression (Figure 2). Most AIM^+^CD4^+^ T cells expressed CD28 but not CD57 (Figure 2A), a marker of T cell senescence (Brenchley et al., 2003). PD-1^high^ cells were observed in memory fractions (Figure 2A), with a portion of them probably representing follicular helper T cells, as previously reported in COVID-19 patients (Mathew et al., 2020). Nevertheless, the distribution of AIM^+^CD4^+^ T cells was similar between the elderly and young (Figure 2A; Supplementary Figure 3). However, PD-1^high^ MP T cells were rare, and CD57^high^ senescent-like T cells were abundant among AIM^+^CD8^+^ T cells, suggesting that SARS-CoV-2 cross-reactive CD8^+^ T cells tend to be senescent rather than exhausted (Figure 2B). Notably, AIM^+^CD8^+^ T cells from the elderly exhibited less distribution in the CCR7^+^CD45RA^+^ NP region and more accumulation in the CD57^high^ region (Figure 2B). Furthermore, IFR4^high^ CD4^+^ T cells were distributed mainly in the memory fraction, whereas IFR4^high^ CD8^+^ T cells accumulated in the NP region (Figures 2A,B, and Supplementary Figure 3). These results suggest that SARS-CoV-2-reactive T cell phenotypes from the young and elderly are similar in CD4^+^ T cells but different for CD8^+^ T cells. Thus, NP CD8^+^ T cells with a high affinity towards SARS-CoV-2 and CD57^+^ senescent SARS-CoV-2-reactive T cell populations were lower and higher, respectively, in the elderly compared with those in the young.

### SARS-CoV-2-Reactive CD4^+^ T Cells Are Preferentially Detected in the CM Fraction With Comparable Numbers in Both Young and Elderly Individuals

Next, we compared the exact frequency and number of each SARS-CoV-2-reactive T cell subpopulation between young and elderly individuals using a manual gating strategy on the flow cytometry data. T cell populations were divided into four fractions based on their expression markers: NP (CD45RA^+^CCD7^+^CD28^+^), CM (CD45RA^+^CCR7^−^), EM (CCR7 CD45RA^−^), and TEMRA (CD45RA^+^CCD7^−^) (Jameson and Masopust, 2018; Thome et al., 2014) (Supplementary Figure 4A). Following the stimulation of PBMCs with CMV peptide, less NP and more MP (EM and TEMRA) AIM^+^ CD4^+^ and CD8^+^ T cells were found in CMV^+^ individuals compared to CMV^−^ individuals (Supplementary Figure 4B). A similar trend was observed in T cells from convalescent patients and uninfected individuals upon SARS-CoV-2 peptide stimulation (Supplementary Figure 4B), consistent with a previous report (Braun et al., 2020).

**FIGURE 3:**
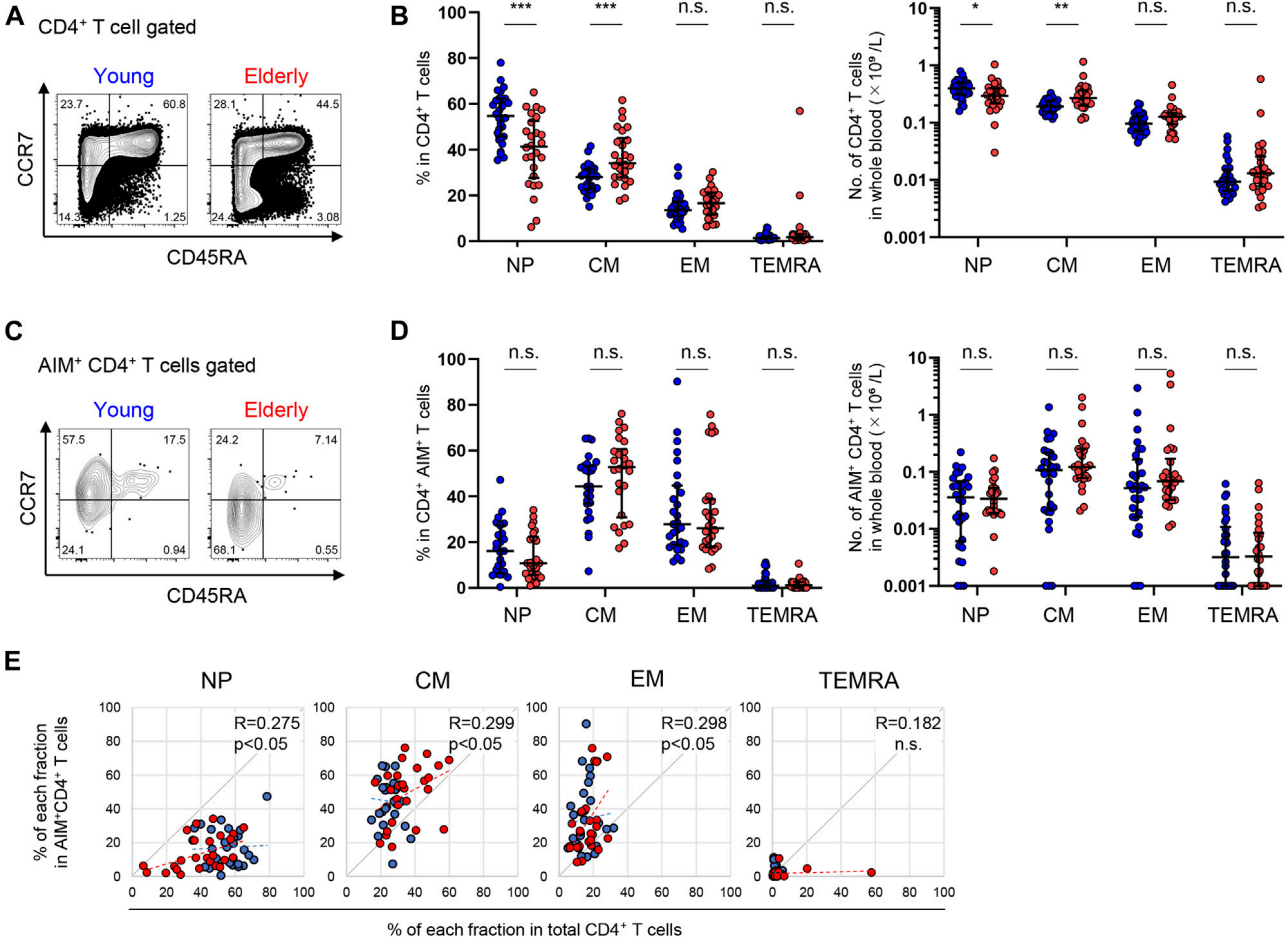
Immunophenotyping of SARS-CoV-2-reactive CD4^+^ T cells from young and elderly individuals. **(A, C)** Representative flow cytometry plots showing the expressions of CD45RA and CCR7 in total CD4^+^ **(A)** and CD4^+^ AIM^+^ (CD137^+^IRF4^+^) **(C)** CD3^+^ T cells after stimulation with the SARS-CoV-2 peptide pool in the AIM assay. The subset definitions and gating strategies are outlined in Supplementary Figures 2A, 4A. Numbers indicate percentages in the drawn gates. **(B, D)** Frequency **(left panels)** and calculated number in whole blood **(right panels)** of NP, CM, EM and TEMRA cells in total CD4^+^ **(B)** and SARS-CoV-2-specific AIM^+^CD4^+^ **(D)** T cells in the young (*n* = 30) and elderly (*n* = 26) cohorts. (**Left panel** of **D**) Samples that a percentage of AIM^+^ cells in CD4^+^ T cells of at least 0.001% are indicated (young, *n* = 27 and elderly, *n* = 26). Data are shown as the median ± IQR. Statistical comparisons across cohorts were performed with the Mann-Whitney test. **(E)** Correlation between the percentage of each fraction in the total CD4^+^ T cell population and of AIM^+^CD4^+^ T cells in the NP, CM, EM, and TEMRA fractions. Correlation coefficients (R) were calculated using the Spearman rank correlation test. Linear approximations are plotted in figures. **(B, D, E)** Each dot represents one donor. Young (blue circles) and elderly (red circles). *p < 0.05; **p < 0.01; ***p < 0.001. n.s., not significant.

Although NP cells represented a dominant fraction of CD4^+^ T cells in both the young and elderly, the percentages [median (interquartile range: IQR)] of the NP [54.7 (15.9)% in the young, 41.3 (21.5)% in the elderly] and CM [28.0 (8.4)% in the young, 34.2 (15.9)% in the elderly] fractions of total CD4^+^ T cells were significantly lower and higher, respectively, in the elderly compared to the young participants, while the EM and TEMRA fractions were comparable between the two groups (Figures 3A,B). A similar trend was observed in the number of cells in each fraction of whole blood between the two groups (Figure 3B). On the other hand, SARS-CoV-2-reactive CD4^+^ T cells were substantially detected in NP fraction in both groups, but were less abundant [16.6 (20.6)% in the young, 10.8 (15.9)% in the elderly] but were highly enriched in the memory fractions (Figures 3C,D; Supplementary Figure 4C), consistent with previous reports (Bacher et al., 2020; Braun et al., 2020; Mateus et al., 2020; Weiskopf et al., 2020). These pre-existing MP SARS-CoV-2-reactive T cells, so-called cross-reactive T cells, were enriched in the CM [44.4 (16.2)% in the young, 52.8 (25.2)% in the elderly] and EM [28.3 (21.9)% in the young, 26.2 (20.4)% in the elderly] fractions (Figures 3C,D). The percentages of NP, CM, and EM cells of the total CD4^+^ T cell population in each individual exhibited positive correlations with their percentages of AIM^+^ cells in each fraction (Figure 3E). However, there were no significant differences in the frequency or number of SARS-CoV-2-reactive CD4^+^ T cells between the young and elderly in any fraction (Figures 3C,D). These results indicate that SARS-CoV-2-reactive T cells are mainly detected in antigen-primed CM fractions in CD4^+^ T cells and that these MP CD4^+^ T cells that were cross-reactive to SARS-CoV-2 were well retained irrespective of age.

### CD4^+^ T Cell Proliferation and Th1 Cytokine Production Upon SARS-CoV-2 Recognition Is Comparable Between Elderly and Young Individuals

**FIGURE 4:**
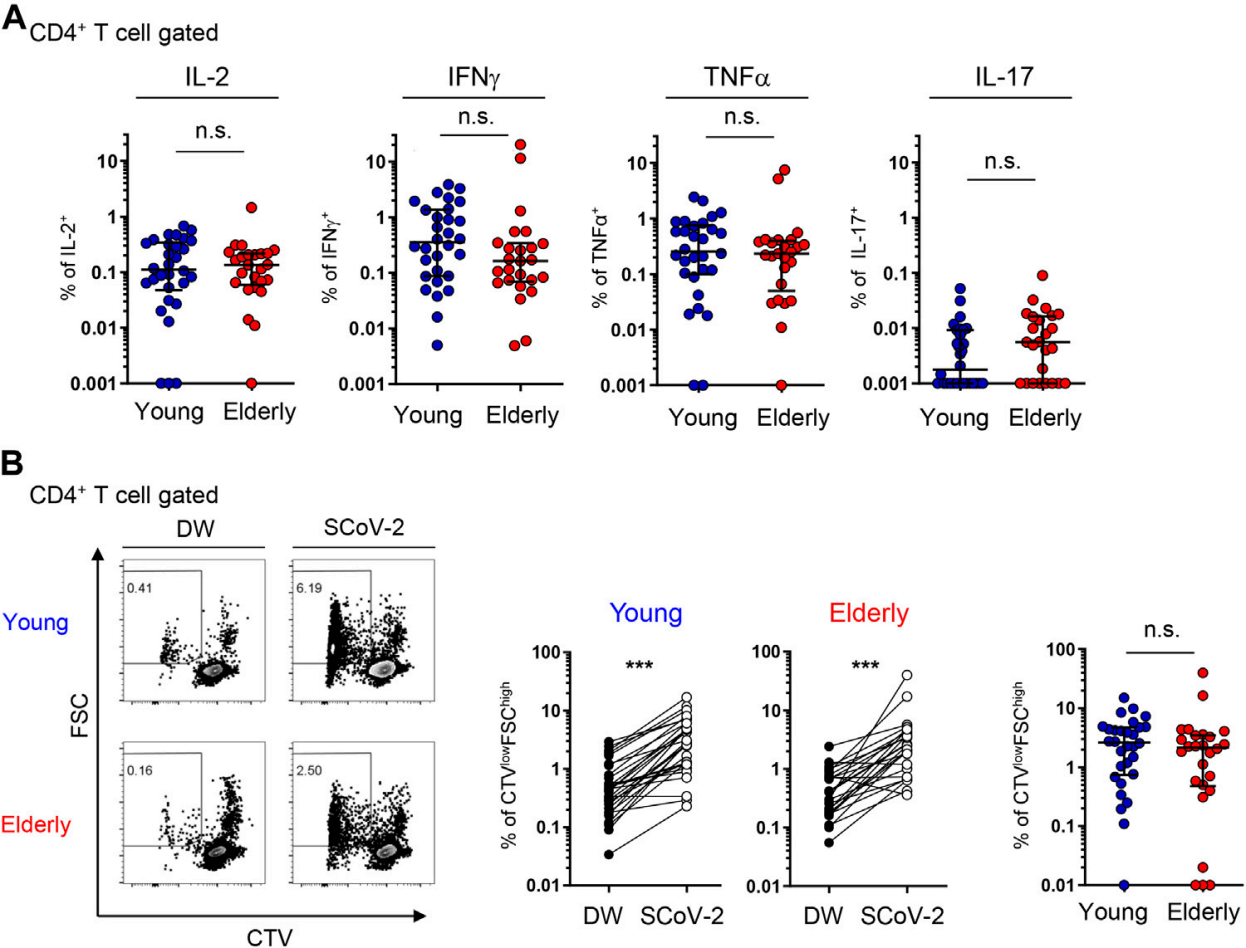
SARS-CoV-2-specific cytokine production and proliferation from young and elderly CD4^+^ T cells. PBMCs from the young (*n* = 30) and elderly (*n* = 25) cohorts were stimulated and cultured with the SARS-CoV-2 peptide pool (SCoV-2) or a negative control (DW) and analyzed after 6 days. One sample from the elderly cohort was excluded because of the low number of PBMCs obtained. **(A)** Percentages of IL-2^+^, IFNγ^+^, TNFα^+^, and IL-17A^+^ cells in the CD4^+^ T cell population from the young (blue circles) and elderly (red circles) cohorts. Each dot represents one donor. Data were background subtracted against DW and are shown as the median ± IQR. **(B)** Representative flow cytometry plots showing CTV and FSC gating of total CD4^+^ T cells. The boxed gates define CTV^low^FSC^high^ cells **(left)**. Percentages of CTV^low^FSC^high^ T cells in CD4^+^ T cells between DW and SARS-CoV-2 peptide pool stimulation (SCoV-2) in the young and elderly cohorts **(middle)**. Frequency of CTV^low^FSC^high^ T cells in CD4^+^ T cells. Data were background subtracted against DW and are shown as the median ± IQR **(right)**. Each dot represents one donor (**left** and **middle**). Pairwise comparisons were performed using Wilcoxon’s test. Statistical comparisons across cohorts were performed using the Mann-Whitney test. ***p < 0.001. n.s., not significant. See Supplementary Figures 5.

Next, we examined the cytokine production and proliferation in response to SARS-CoV-2 peptide stimulation to assess the functionality and polarization of the SARS-CoV-2-specific CD4^+^ T cell response. Previous reports have shown that SARS-CoV-2 peptide stimulation significantly induces IFNγ production by CD4^+^ T cells from unexposed individuals (Braun et al., 2020; Grifoni et al., 2020; Le Bert et al., 2020; Mateus et al., 2020; Meckiff et al., 2020; Sekine et al., 2020; Weiskopf et al., 2020) and that the Th1 response is associated with an effective resolution of infection and symptoms in COVID-19 (Chen et al., 2020; Neidleman et al., 2020). CTV-labeled PBMCs were cultured with the peptide pools, and cytokine expression and proliferation estimated by CTV dilution were analyzed at days 3 and 6. The gating strategy is shown in Supplementary Figure 5A. CD4^+^ T cells expressing intracellular Th1 cytokines, including IFNγ, TNFα, and IL-2, were detected in both the young and elderly (Figure 4A and Supplementary Figures 5A,C), and these cytokines were significantly detected in culture supernatants (Supplementary Figure 5D). Th2 cytokines, such as IL-4 and IL-5, as well as IL-17, were produced at markedly low levels (Supplementary Figure 5D), consistent with a previous report (Grifoni et al., 2020). Although the frequencies of intracellular IFNγ^+^ cells tended to be lower and those of IL-17^+^ cells tended to be higher in the elderly, there was no statistically significant difference between elderly and young participants (Figure 4A). Indeed, most participants in the two groups showed similar frequencies (Figure 4A). Furthermore, no significant difference in the percentages of CTV-diluted FSC^high^ proliferated T cells after peptide stimulation between the young and elderly was observed (Figure 4B), suggesting that SARS-CoV-2-reactive CD4^+^ T cells have comparable proliferation capacity in the two populations. Cytokine-producing cells were mainly detected in the CTV^low^ cell fraction of both groups, but the percentages of IFNγ^+^ cells tended to be lower in the elderly compared to the young (Supplementary Figure 5E). These data strongly suggest that SARS-CoV-2-reactive CD4^+^ T cells in the elderly can proliferate and produce helper cytokines upon SARS-CoV-2 recognition despite the tendency for less IFNγ production.

### Elderly Individuals Harbor Less Naïve and More TEMRA SARS-CoV-2-Reactive CD8^+^ T Cells

**FIGURE 5:**
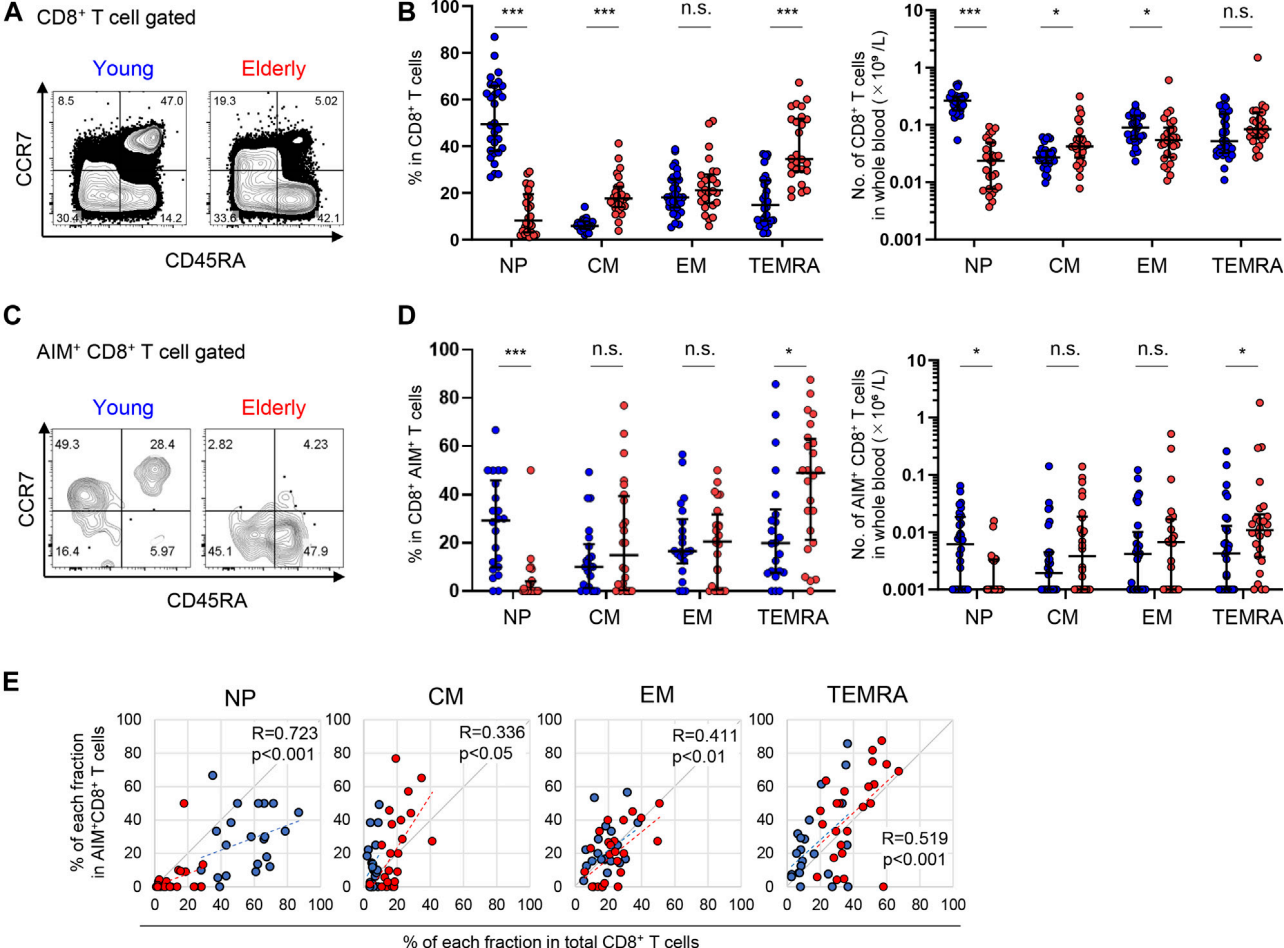
Immunophenotyping of SARS-CoV-2-reactive CD8^+^ T cells from young and elderly individuals. **(A**,**C)** Representative flow cytometry plots showing the expression of CD45RA and CCR7 among total CD8^+^ **(A)** and CD8^+^CD137^+^IRF4^+^ **(C)** T cells after stimulation with the SARS-CoV-2 peptide pool in the AIM assay. Subset definitions and gating strategies are outlined in Supplementary Figures 2A, 4A. Numbers indicate population percentages in the gates. **(B**,**D)** Frequency (left panels) and calculated total number in the whole blood sample **(right panels)** of NP, CM, EM, and TEMRA cells in total CD8^+^ **(B)** an SARS-CoV-2-reactive AIM^+^ CD8^+^ **(D)** T cells in the young (*n* = 30) and elderly (*n* = 26) cohorts. (**Left panel** of **D**) Samples that a percentage of AIM^+^ cells in CD8^+^ T cells of at least 0.001% are indicated (young, *n* = 22 and elderly, *n* = 24). Data are shown as the median ± IQR. Statistical comparisons across cohorts were performed using the Mann-Whitney test. **(E)** Correlation between percentages of each fraction in total CD8^+^ T cells and of AIM^+^CD8^+^ T cells in the NP, CM, EM, and TEMRA fractions. Correlation coefficients (R) were calculated with the Spearman rank correlation test. Linear approximations are plotted in figures. **(B,D,E)** Each dot represents one donor. Young (blue circles) and elderly (red circles). *p < 0.05; **p < 0.01; ***p < 0.001. n.s., not significant.

A similar analysis was performed on CD8^+^ T cells. Compared to CD4^+^ T cells, the percentage of NP cells among total CD8^+^ T cells was significantly lower in the elderly [8.2 (15.2)%] compared to the young [49.5 (26.7)%]; however, the percentages of CM and TEMRA cells were higher in the elderly [17.7 (7.8)% and 34.6 (21.9)%, respectively] compared to the young [5.9 (3.0)% and 14.9 (17.0)%, respectively] (Figures 5A,B). Moreover, the number of NP cells was remarkably fewer in the elderly (Figure 5B). On the other hand, SARS-CoV-2-reactive AIM^+^ CD8^+^ T cells were more diffusely distributed in each phenotype with significant individual variability (Figures 5C,D). Notably, elderly individuals had a significantly lower frequency and number of NP SARS-CoV-2-reactive CD8^+^ T cells compared with the young [28.6 (36.2)% and 6.19 (1.7) × 10^3^ cells/L in young; 0.0 (7.9)% and 0.0 (3.3) × 10^3^ cells/L in elderly] (Figures 5C,D). On the other hand, the percentages as well as numbers of MP (cross-reactive) SARS-CoV-2-reactive CD8^+^ T cell were significantly higher in the elderly (Supplementary Figure 4C). Furthermore, SARS-CoV-2-reactive CD8^+^ T cells were most frequently observed in the TEMRA fraction from the elderly [49.0 (36.0)% and 10.9 (14.3) × 10^3^ cells/L), and their prevalence was significantly higher in the elderly than that in the young [21.1 (36.0)% and 4.3 (11.3) × 10^3^ cells/L] (Figures 5C,D). The percentages of each phenotype of CD8^+^ T cells exhibited weak correlations with the percentages of AIM^+^ cells in each fraction (Figure 5E). These data indicate that young individuals have more SARS-CoV-2-reactive NP CD8^+^ T cells than do elderly individuals. Additionally, MP CD8^+^ T cells cross-reactive to SARS-CoV-2 in the elderly were biased towards TEMRA cells, which exhibit highly differentiated phenotypes (Thome et al., 2014; Jameson and Masopust, 2018).

### Accumulation of Senescent SARS-CoV-2-Reactive CD8^+^ T Cells in Elderly Individuals

**FIGURE 6:**
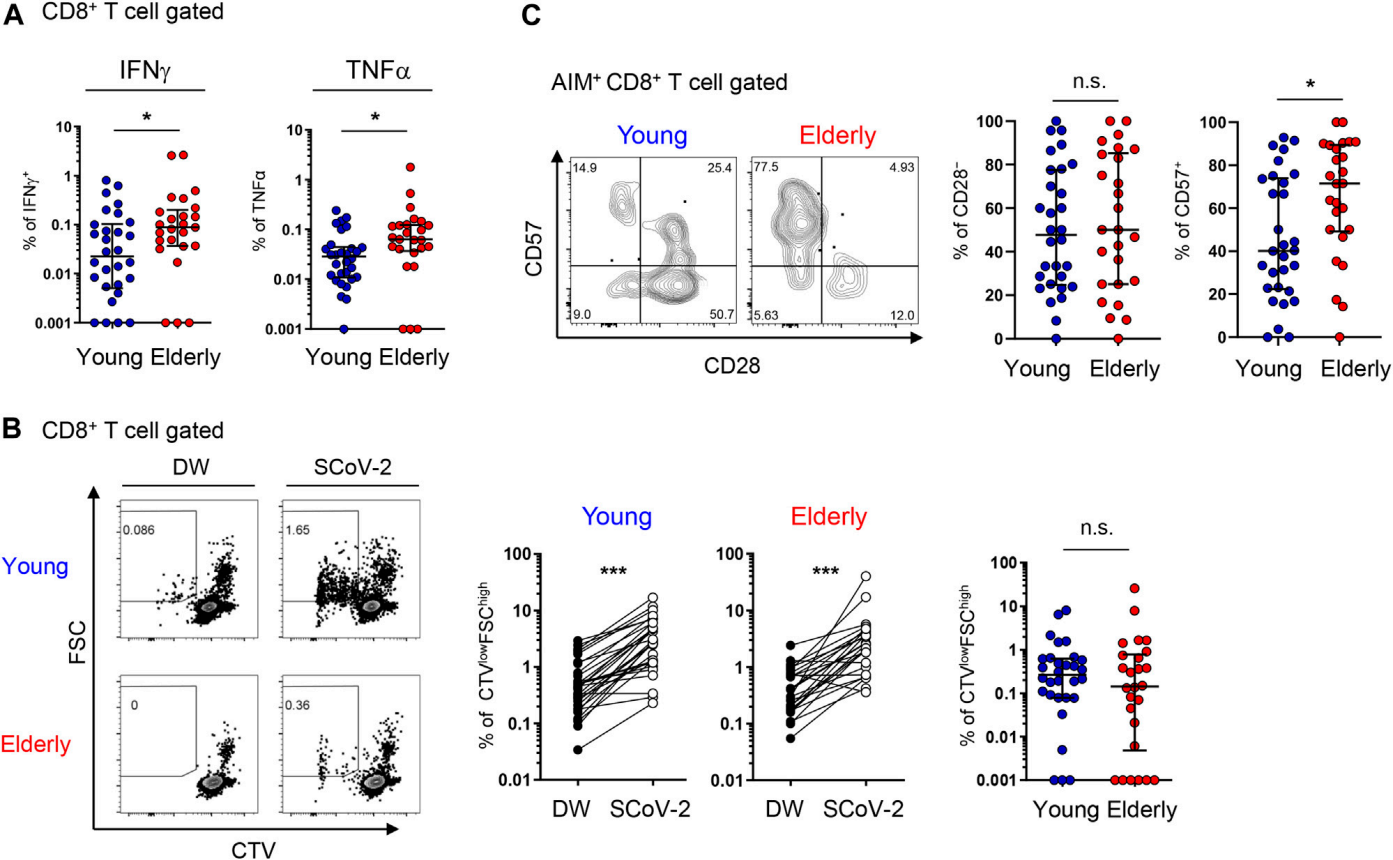
SARS-CoV-2-specific cytokine production and proliferation from young and elderly CD8^+^ T cells. PBMCs from the young (*n* = 30) and elderly (*n* = 25) cohorts were stimulated and cultured with the SARS-CoV-2 peptide pool (SCoV-2) or negative control (DW) and analyzed after 6 days. One sample from the elderly cohort was excluded because of the low number of PBMCs obtained. **(A)** Percentages of IFNγ^+^ and TNFα^+^ cells in CD8^+^ T cells from the young (blue circles) and elderly (red circles) cohorts. Data were background subtracted against DW and are shown as the median ± IQR. **(B)** Representative flow cytometry (FCM) plots showing CTV and FSC gating of total CD8^+^ T cells. Boxed gates define CTV^low^FSC^high^ cells **(left)**. Percentages of CTV^low^FSC^high^ T cells in CD8^+^ T cells between the DW and SARS-CoV-2 peptide pool stimulation in young and elderly cohorts. **(middle)**. Percentages of CTV^low^FSC^high^ T cells in CD8^+^ T cells. Data were background subtracted against DW and are shown as the median ± IQR **(right)**. **(C)** Representative FCM plots showing the expression of CD57 and CD28 in AIM^+^ CD8^+^ T cells in the AIM assay. Numbers indicate percentages in the drawn gates **(left)**. Percentages of CD28^−^ and CD57^+^ cells in SARS-CoV-2-specific AIM^+^ CD8^+^T cells from young and elderly cohorts. Data are shown as the median ± IQR (right). **(A**-**C)** Each red or blue dot represents one donor. Pairwise comparisons were performed using Wilcoxon’s test. Statistical comparisons across cohorts were performed using the Mann-Whitney test. *p < 0.05, ***p < 0.001. n.s., not significant. See Supplementary Figure 6.

Regarding functionality, CD8^+^ T cells from the elderly showed higher proportions of IFNγ^+^ and TNFα^+^ cells compared to those from young participants after 6 days of culture with SARS-CoV-2 peptides (Figure 6A and Supplementary Figures 6A,B). The percentage of CTV-diluted FSC^high^ cells did not differ between the two populations (Figure 6B). Considering that the percentage in AIM^+^ CD8^+^ T cells was higher in the elderly than in young individuals (Figure 1C), SARS-CoV-2-reactive CD8^+^ T cells from the elderly may have a lower proliferative capacity. In contrast to CD4^+^ T cells, the percentage of cytokine^+^ cells was higher in less proliferated CTV^high^ cells, with this tendency more obvious in the elderly (Supplementary Figures 6C). These features may be consistent with the elderly SARS-CoV-2 reactive CD8^+^ T cells being highly enriched in the TEMRA fraction (Figure 5D).

TEMRA cells include CD28^−^ terminally differentiated T cells with compromised proliferation capacities, as well as CD57^+^ senescent T cells with a high potential for inflammatory cytokine production (Brenchley et al., 2003; Henson et al., 2015). Therefore, we further assessed CD28 and CD57 expression in AIM^+^ T cells. We found that the percentage and number of CD57^+^ cells, but not CD28^−^ cells, among AIM^+^ CD8^+^ T cells from the elderly were significantly higher than those from young participants (Figure 6C) and that CD28^−^ or CD57^+^ AIM^+^ CD4^+^ T cells were rare (Supplementary Figure 7). These findings suggest that senescent SARS-CoV-2-reactive CD8^+^ T cells with compromised proliferation capacity and high effector functions tended to accumulate in the elderly.

### Young CMV Seropositive Individuals Exhibit a Higher Proportion of Senescent SARS-CoV-2-Reactive CD8^+^ T Cells Than Young Seronegative Individuals

**FIGURE 7:**
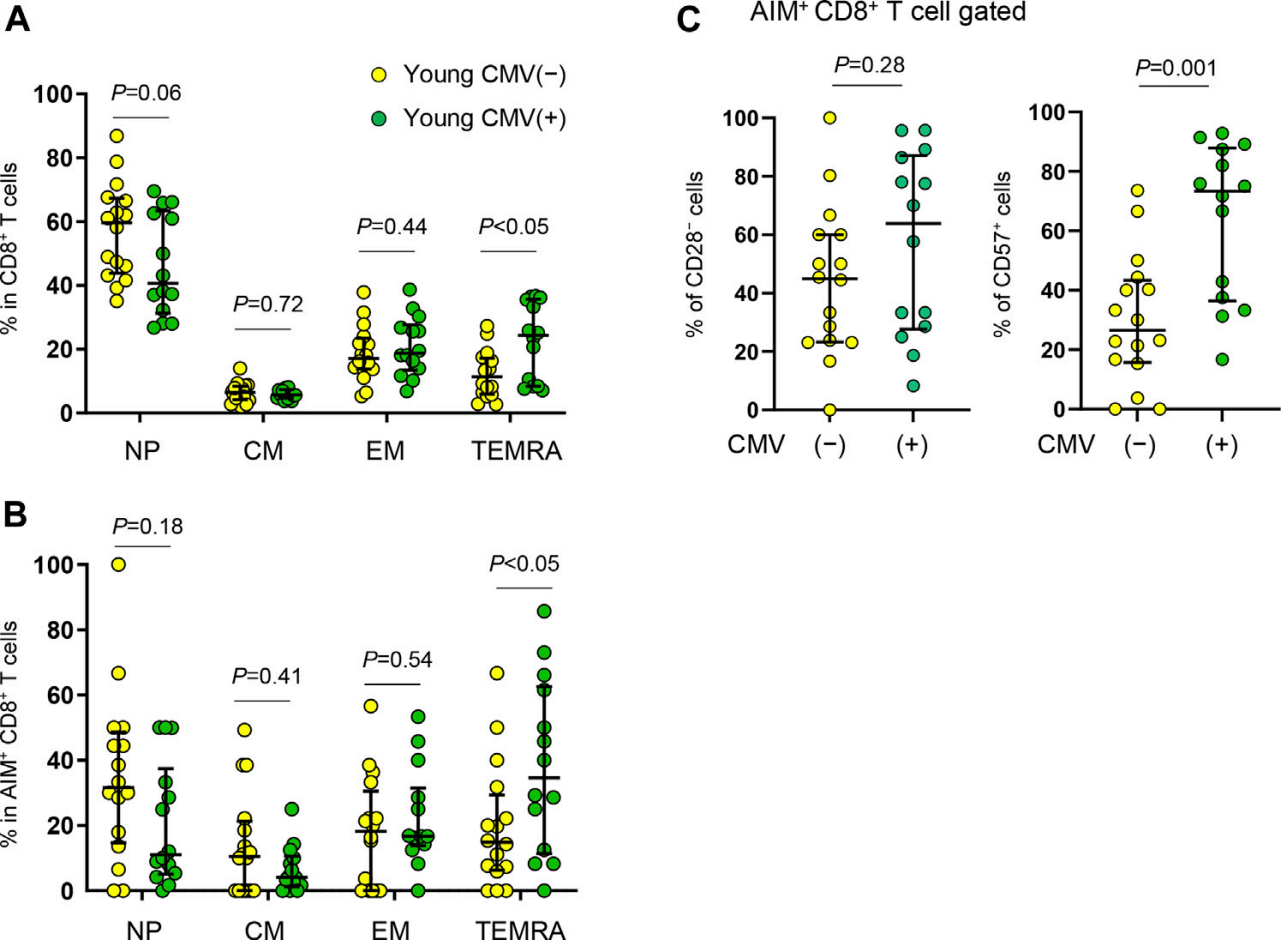
Immunophenotyping of SARS-CoV-2-reactive CD8^+^ T cells from young CMV^+^ or CMV^−^ individuals. **(A,B)** Percentages of NP, CM, EM and TEMRA cells in total CD8^+^ **(A)** or in SARS-CoV-2-reactive AIM^+^CD8^+^ T cells **(B)** from CMV seronegative (CMV(−)) and CMV seropositive (CMV(+)) individuals. **(C)** Frequency of CD28^−^ and CD57^+^ cells among SARS-CoV-2-specific AIM^+^CD8^+^ T cells from CMV^+^ (*n* = 14) and CMV^−^ (*n* = 16) individuals. Each dot represents one donor. Data are shown as the median ± IQR. Statistical comparisons across cohorts were performed using the Mann-Whitney test.

We noticed that young individuals are divided into two groups based on the percentage of CD57^+^ cells (high, > 60%, *n* = 11 and low, < 50%, *n* = 19) in SARS-CoV-2-reactive CD8^+^ T cells (Figure 6C). All elderly individuals were CMV^+^, but approximately half (46.7%) of young individuals were CMV^+^ (Table 1). Because CMV infection markedly affects CD8^+^ T cell homeostasis and the generation of senescent T cells (Nikolich-Zugich, 2008; Wertheimer et al., 2014; Klenerman and Oxenius, 2016), we re-analyzed the AIM^+^ cell phenotypes from young individuals as CMV^+^ (*n* = 14) and CMV^−^ (*n* = 16). Consistent with previous studies (Nikolich-Zugich, 2008; Wertheimer et al., 2014), young CMV^+^ individuals tended to have proportionally less NP and more TEMRA CD8^+^ T cells (Figure 7A). Importantly, young CMV^+^ individuals showed significantly more TEMRA as well as CD57^+^ senescent SARS-CoV-2-reactive CD8^+^ T cells (Figures 7B,C). SARS-CoV-2-reactive TEMRA CD4^+^ T cells were rare, and no marked difference was observed in CD57^+^CD4^+^ T cells (Supplementary Figure 8). These results indicate that CMV infection affects the SARS-CoV-2-reactive CD8^+^ T cell frequency in young individuals.

## Discussion

Our understanding of T cell immunity against SARS-CoV-2 is growing, but the difference in T cell responses between young and elderly individuals remains poorly understood. Japan is well known for the longevity of its citizens, and it is also known for a low COVID-19 death rate (WHO Coronavirus disease (COVID-19) Dashboard, https://covid19.who.int). Our results suggest that approximately 30–50% of SARS-CoV-2 antibody seronegative (presumably uninfected) Japanese individuals have significant levels of pre-existing SARS-CoV-2-reactive CD4^+^ T cells. Although simultaneous analysis using the same assays, markers, and gating strategies is required for a precise comparison, the percentage of individuals with significant levels of pre-existing SARS-CoV-2-reactive T cells was comparable to that in other countries (Braun et al., 2020; Grifoni et al., 2020; Le Bert et al., 2020; Mateus et al., 2020; Sekine et al., 2020; Thieme et al., 2020; Weiskopf et al., 2020).

Despite the significant reduction in thymic T cell production after adolescence, NP CD4^+^ T cells were well maintained in the elderly and comprised the largest population in both the young and the elderly. However, NP AIM^+^ CD4^+^ SARS-CoV-2-reactive T cells comprised less than 20% of total AIM^+^ CD4^+^ T cells, and SARS-CoV-2-reactive CD4^+^ T cells were mainly detected in the CM and EM fractions, consistent with studies from the U.S. and Germany (Bacher et al., 2020; Braun et al., 2020; Mateus et al., 2020; Weiskopf et al., 2020). This trend was similar in both young and elderly Japanese individuals. These findings strongly suggest that a large proportion of CD4^+^ T cells exhibiting SARS-CoV-2 reactivity is already primed, presumably by the common cold coronaviruses (Bacher et al., 2020; Braun et al., 2020; Mateus et al., 2020) or other unrelated antigens (Bacher et al., 2020) and that these cells are maintained in individuals unexposed to SARS-CoV-2 as MP cells that can cross-react with SARS-CoV-2. The SARS-CoV-2-reactive CM CD4^+^ T cells circulate in secondary lymphoid tissues and may quickly respond to actual SARS-CoV-2 infection (Mahnke et al., 2013; Jameson and Masopust, 2018). The phenotypic characteristics of pre-existing SARS-CoV-2-reactive T cells may be consistent with the observation that seroconversion for anti-SARS-CoV-2 IgM and IgG occurs simultaneously in some COVID-19 patients (Long et al., 2020). Regarding T cell functions, strong Th1 cell responses are associated with low disease severity (Chen et al., 2020; Neidleman et al., 2020; Rydyznski Moderbacher et al., 2020). Although the results did not reach statistical significance, probably due to individual variability and low sample number, IFNγ production appeared to be lower in the elderly, which is consistent with a recent report showing no significant upregulation of IFNγ production in the elderly after the first dose of SARS-CoV-2 vaccine BNT162b2 (Collier et al., 2021). Overall, however, we found no significant difference in Th1, Th2, and Th17 cytokines, nor other inflammatory cytokine production between the young and elderly. Moreover, CD4^+^ T cells derived from young and elderly subjects showed a similar proliferation capacity, at least in short-term culture, upon SARS-CoV-2-derived peptide stimulation. Thus, little functional abnormality was detected in SARS-CoV-2-reactive CD4^+^ T cells from most healthy elderly individuals. Therefore, CD4^+^ T cell responses can be induced early after SARS-CoV-2 infection and/or vaccination in individuals with sufficient SARS-CoV-2-reactive CD4^+^ T cells irrespective of age in a Japanese cohort.

It has been suggested that the poor disease outcome in aged individuals may be related to a decreased CD8 T cell response (Rydyznski Moderbacher et al., 2020; Takahashi et al., 2020; Zhang et al., 2020). In contrast to previous reports suggesting little evidence of cross-reactive CD8^+^ MP T cells (Lipsitch et al., 2020), our findings provide clear evidence of pre-existing SARS-CoV-2 MP as well as NP CD8^+^ T cells. This may be attributed to the use of AIM combination in the present study, which enabled the detection of antigen-specific T cells in a very low background (Wu et al., 2019), and/or regional differences, which may be affected by previous exposure to common-cold coronaviruses and/or HLA haplotypes. Nonetheless, our results demonstrated a significant reduction in the number of total NP CD8^+^ T cells and SARS-CoV-2-reactive NP CD8^+^ T cells in the elderly. Furthermore, the percentage of the NP fraction of the total CD8^+^ T cells was positively correlated with that of NP SARS-CoV-2-reactive CD8^+^ T cell fraction. Therefore, the frequency of SARS-CoV-2-reactive NP T cells in each individual may be roughly predictable from the fraction of NP CD8^+^ T cells.

NP T cells ensure reactivity against a broad range of unexperienced antigens. They also exhibit high proliferative capacity and generate effectors as well as memory T cells (Sallusto et al., 1999). An opt-SNE analysis of AIM^+^CD8^+^ T cells indicated that IRF4^high^ cells, which may show high affinity/avidity to SARS-CoV-2, were preferentially detected in the NP fraction, suggesting that TCRs of cross-reactive MP CD8^+^ T cells may show low affinity/avidity to SARS-CoV-2, as previously reported in CD4^+^ T cells (Bacher et al., 2020). In general, TCR signaling can scale the magnitude of T cell expansion and thus the number of effector T cells (Zehn et al., 2009). Additionally, a previous report suggested a correlation between fewer naïve CD8 T cells and the severity of COVID-19 (Rydyznski Moderbacher et al., 2020). Furthermore, T cell immunity recognizing multiple epitopes, presumably assured by NP SARS-CoV-2-reactive T cells, is associated with mild symptoms of COVID-19 (Nelde et al., 2021). Taken together, the low frequency and number of NP SARS-CoV-2-reactive CD8^+^ T cells in the elderly may be a factor for their low anti-viral immunity.

T cells are also assumed to play a pathogenic role in COVID-19. Indeed, patients with a deficiency in acquired immunity, including patients with HIV or congenital B cell deficiency, are not categorized as a high-risk group (Banerjee et al., 2020; Fung and Babik, 2021). Damage to lung tissue by highly differentiated senescent-like T cells in COVID-19 patients has also been discussed (Akbar and Gilroy, 2020). While some studies have claimed little evidence to support this hypothesis (Rydyznski Moderbacher et al., 2020), others have suggested that the hyperactivation of differentiated effector memory T cells could play a role in the immunopathogenesis in critical patients (De Biasi et al., 2020; Thieme et al., 2020). Although the duration for which memory T cells against common cold coronaviruses are maintained is not well understood, our results indicated that the number of SARS-CoV-2-reactive T cells is comparable to, or even relatively higher, in the elderly than in the young. Notably, SARS-CoV-2 cross-reactive CD8^+^ T cells in the elderly were highly biased toward highly differentiated TEMRA cells with high effector cytokine production but low proliferation capacity. A previous report indicated that SARS-CoV-2-reactive CD8^+^ T cells from convalescent patients with mild COVID-19 were predominantly TEMRA cells expressing CD28, suggesting a protective role (Neidleman et al., 2020). However, our study revealed that TEMRA cells that accumulated in the elderly lacked CD28 and/or expressed CD57, strongly suggesting that they undergo replicative senescence and are prone to antigen-induced apoptotic cell death (Brenchley et al., 2003). Therefore, our results suggest the intriguing possibility that senescent SARS-CoV-2-reactive CD8^+^ T cells may exhibit adequate functionality during a short period after activation but have low proliferation capacity, eventually undergoing apoptosis and cell death. This may provide a mechanism for the mild symptoms observed in the early phase of infection, but eventually, the disease progresses to severe symptoms in the later phase owing to defective viral clearance in the elderly. The correlation between disease severity upon SARS-CoV-2 infection and pre-existing TEMRA CD8^+^ cells expressing CD57 and/or lacking CD28 expression should be investigated in the future.

In addition to age-related differences, we observed significant individual variability in the frequencies and phenotypes of pre-existing SARS-CoV-2-reactive T cells within the same age groups. This variability may be due, in part, to differences in recent exposures to common cold coronaviruses as well as thymic activity that assures naïve T cell production and is heavily affected by various stresses and even dietary lifestyle (Chaudhry et al., 2016). Unexpectedly, our results also suggested that CMV infection could be a factor for the individual variability in SARS-CoV-2-reactive T cell phenotype. Although the precise mechanism underlying CMV infection-induced SARS-CoV-2-recognizing T cell senescence is unknown, some reports indicated that cross-reaction occurs more frequently than initially thought. For example, HIV-reactive MP T cells have been detected even in HIV-uninfected individuals, and these cells are reactive towards other microbial peptides, suggesting that cross-reactive T cells can be generated by exposure even to unrelated environmental antigens (Su et al., 2013). Therefore, one possibility would be that a portion of CMV-reactive senescent T cells could cross-react with SARS-CoV-2. Furthermore, in the context of COVID-19, CMV could contribute to poor disease outcomes not only by accelerating the aging of the T cell compartment (Nikolich-Zugich, 2008; Wertheimer et al., 2014; Klenerman and Oxenius, 2016), but also through tissue damage by CMV reactivation under the lymphopenic condition and by a TCR-independent activation of senescent T cells (Akbar and Gilroy, 2020; Soderberg-Naucler, 2021). Importantly, a recent study found that the expansion of CD57^+^KLRG1^+^ T cells in CMV^+^ young adults is associated with a reduced Ebola vaccine response (Bowyer et al., 2020), suggesting that CMV infection could also have negative impacts on vaccination efficacy. However, some reports indicated a positive contribution of CMV to the immune reaction by broadening the mobilized TCR repertoire (Smithey et al., 2018) and by upregulating the basal activation status of innate immunity (Barton et al., 2007). Indeed, one report indicated that CMV-seropositive young adults exhibit enhanced antibody responses to influenza vaccination (Furman et al., 2015). Whether CMV infection and/or CMV IgG titers are associated with disease outcomes of post-SARS-CoV-2 infection as well as the efficacy of SARS-CoV-2 vaccination remains to be determined.

In summary, we identified the quantitative and qualitative differences in SARS-CoV-2-reactive T cells between young and elderly unexposed individuals. Although we found no significant differences in CD4^+^ T cells, we found fewer NP and more TEMRA CD8^+^ T cells, especially senescent SARS-CoV-2-reactive CD8^+^ T cells, in the elderly. These findings shed light on the age-related differences and individual variations in pre-existing T-cell phenotypes before exposure to SARS-CoV-2 as factors for different COVID-19 severities, an issue that is key to developing efficient therapeutic strategies as well as predicting vaccine efficacy.

## Limitation of Study

This study reported less NP and more senescent SARS-CoV-2-reactive CD8^+^ T cell populations in an older unexposed Japanese cohort. Our findings provide correlative evidence for the poor outcomes in elderly COVID-19 patients, but the causal link and functional significance of cross-reactive T cells remain unclear. Since lifespan, health, and infection history differ between countries, further analysis will be required using other geographical cohorts to generalize these findings. The risk for severe COVID-19 and death begins to rise strongly from around 55 years of age. It remains to be determined whether the CD8^+^ T cells from unexposed donors of middle ages are more similar to those of 20-year-olds or 70-year-olds. Additionally, because NP T cells are defined as CCR7^+^CD45RA^+^ cells, stem cell memory T cells, if any, were not evaluated. Peptide pools containing major SARS-CoV-2 epitopes (S-, N-, and M-proteins) were used for the AIM assay, but target epitopes and avidity may affect the consequence of the cross-reactions, especially those of CD4 T cells (Bacher et al., 2020; Braun et al., 2020). Differences in the recognized epitopes between SARS-CoV-2-reactive T cells from the young and elderly were not addressed, and T cells recognizing other epitopes were not evaluated. Our functional analysis was limited to proliferation and cytokine production in a short-term culture due to limited sample availability. Broader analysis, such as killing activity, long-term maintenance, and recruitment to inflammatory sites after antigen stimulation, would provide a more detailed characterization of SARS-CoV-2-reactive T cells from the young and elderly. Other factors affecting T cell functionality, including antigen presentation capacity, were not evaluated in this study. Blood samples used in this study were collected between July and September 2020 during the COVID-19 pandemic. Although all samples were SARS-CoV-2 Ig negative, and the prevalence of SARS-CoV-2 antibodies in June 2020 was less than 0.2% in metropolitan areas in Japan (https://www.mhlw.go.jp/stf/seisakunitsuite/bunya/0000121431_00,132.html), we cannot exclude the possibility that convalescents with no symptoms or no SARS-CoV-2 antibody production were included in the study cohort.

## Data Availability

The original contributions presented in the study are included in the article/Supplementary Material, further inquiries can be directed to the corresponding author.
