# Supplementary material for: Aging and CMV Infection Affect Pre-existing SARS-CoV-2-Reactive CD8+ T Cells in Unexposed Individuals
Source: Front Aging. 2021 Aug 10;2:719342. doi: 10.3389/fragi.2021.719342 (PMC9261342; doi:10.3389/fragi.2021.719342)
Supplement: Supplementary file 1 [file DataSheet1.pdf]

Supplementary Material

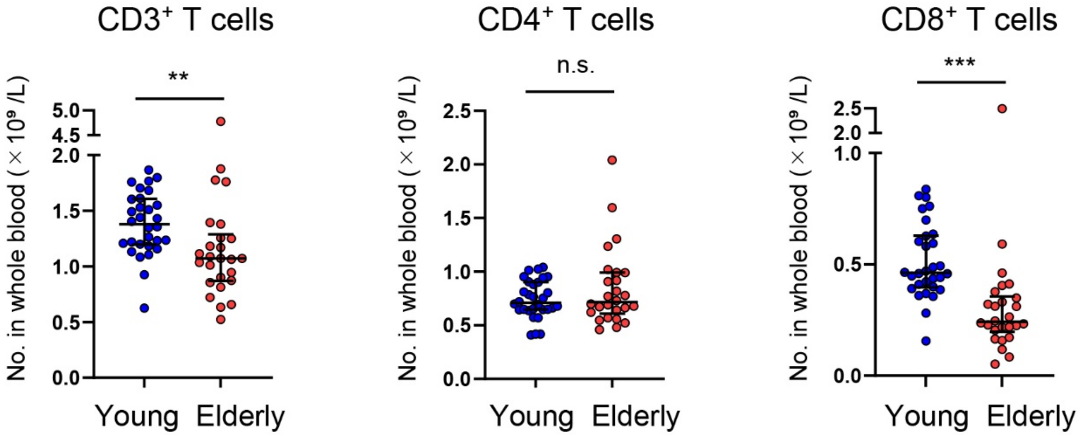

Supplementary Figure 1. Calculation of T cell number

Absolute $CD3^+$, $CD4^+$ and $CD8^+$ T cell numbers in whole blood ($\times 10^9$ cells/L) from the young (n = 30) and elderly (n = 26) cohorts were calculated by multiplying the percentages by the number of total lymphocytes determined from complete blood counts. Statistical comparisons between cohorts were performed using the Mann-Whitney test. $^{**}p < 0.01$; $^{***}p < 0.001$. n.s., not significant.

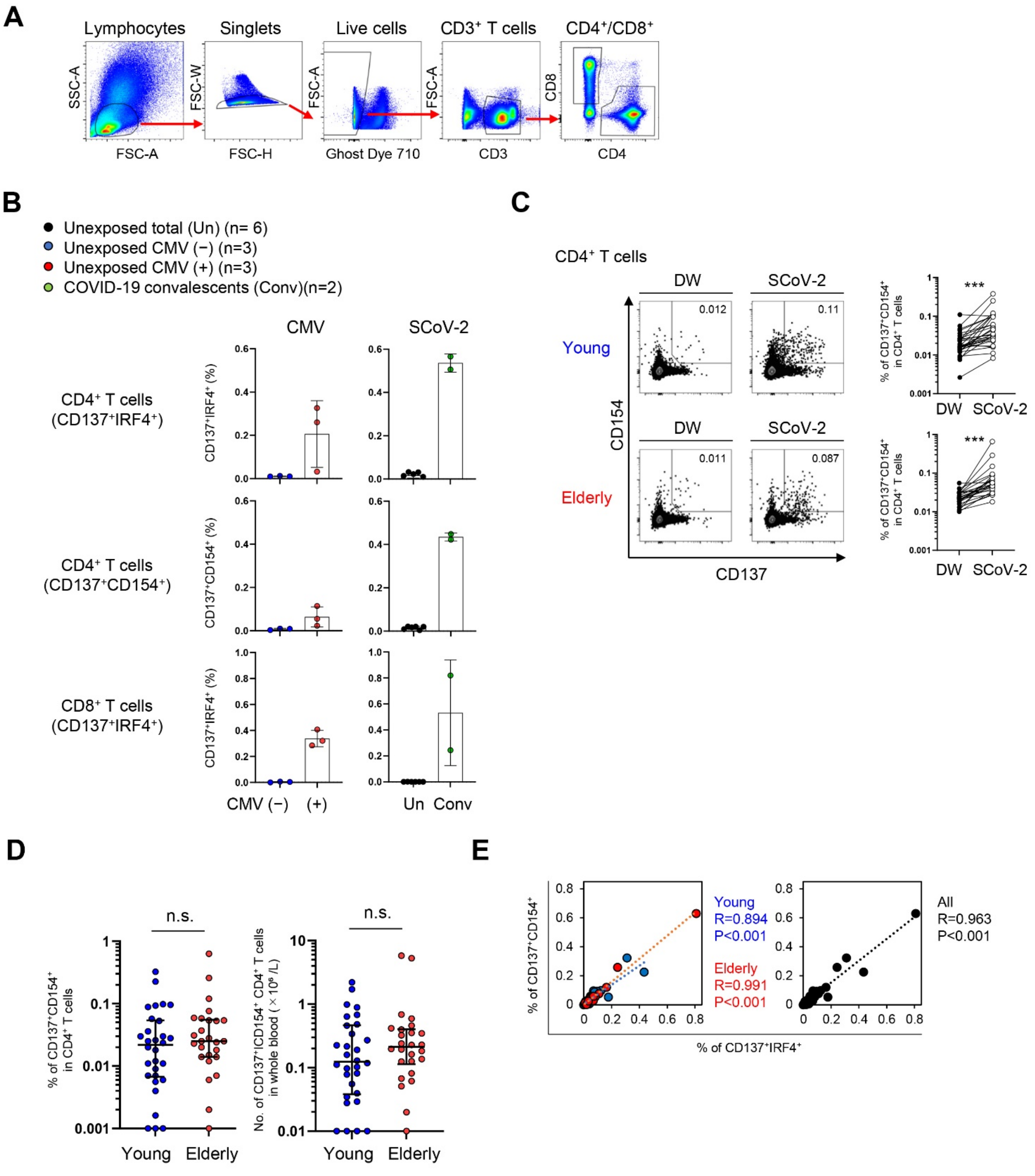
A
Lymphocytes
Singlets
Live cells
CD3+ T cells
CD4+/CD8+
SSC-A
FSC-A
FSC-W
FSC-H
Ghost Dye 710
CD3
CD8
CD4
B
Unexposed total (Un) (n= 6)
Unexposed CMV (−) (n=3)
Unexposed CMV (+) (n=3)
COVID-19 convalescents (Conv)(n=2)
CMV
SCoV-2
CD4+ T cells (CD137+IRF4+)
CD4+ T cells (CD137+CD154+)
CD8+ T cells (CD137+IRF4+)
CD137+IRF4+ (%)
CD137+CD154+ (%)
CMV (−) (+)
Un Conv
C
CD4+ T cells
DW
SCoV-2
Young
Elderly
CD154
CD137
0.012
0.11
0.011
0.087
% of CD137+CD154+ in CD4+ T cells
***
D
n.s.
% of CD137+CD154+ in CD4+ T cells
No. of CD137+CD154+ CD4+ T cells in whole blood (×10⁶/L)
Young
Elderly
E
% of CD137+CD154+
% of CD137+IRF4+
Young
R=0.894
P<0.001
Elderly
R=0.991
P<0.001
All
R=0.963
P<0.001

Supplementary Figure 2. Gating strategy and feasibility of AIMs

(A) Representative gating strategy to detect $CD3^+$ T cells, $CD4^+$ T cells and $CD8^+$ T cells in the AIM assay. Briefly, lymphocytes were gated out of all events, and doublet exclusion was performed. Live cells were gated as Ghost Dye^{TM} Red 710^-. T cells were then gated as $CD3^+$ and further subdivided into $CD4^+$ and $CD8^+$ populations. $CD4^+$ and $CD8^+$ T cells were further analyzed with the additional markers described in Supplementary Table 1. **(B)** PBMCs isolated from CMV^- SARS-CoV-2 unexposed individuals (n = 3), CMV^+ SCoV-2 unexposed individuals (n = 3), and COVID-19 convalescents without the CMV test (ND; not determined) (n = 2) were stimulated for 20 hours with DW or overlapping peptides containing CMV-pp65 sequences (CMV) or SARS CoV-2 S, N, and M protein sequences (SCoV-2). The percentages of AIM^+ ($CD137^+IRF4^+$ or $CD137^+CD154^+$) $CD4^+$ and AIM^+ ($CD137^+IRF4^+$) $CD8^+$ T cells are shown. Data were background subtracted against DW and are shown as the mean ± SD. **(C)** Representative FCM plots showing the expression of CD154 and CD137 in the $CD4^+$ T cell population after stimulation with the SARS-CoV-2 peptide pool (SCoV-2) or negative control (DW) in the AIM assay. Numbers indicate percentages in the drawn gates (left). Percentages of AIM^+ ($CD137^+CD154^+$) cells in $CD4^+$ T cells between DW and SARS-CoV-2 peptide pool stimulation for young (top) and elderly (bottom) cohorts (right). **(D)** Frequency (left) and calculated number in whole blood (right) of AIM^+ ($CD137^+CD154^+$) T cells in $CD4^+$ T cells from the young and elderly cohorts. Data were background subtracted against DW and are shown as the median ± IQR. Pairwise comparisons were performed with the Wilcoxon test. Statistical comparisons across cohorts were performed with the Mann-Whitney test. **(E)** Correlation between the percentages of $CD137^+IRF4^+$ cells and $CD137^+CD154^+$ cells in the total $CD4^+$ T cell population. Correlation coefficients (R) were calculated using the Spearman rank correlation test. Linear approximations are plotted in the figures. (C, D, E) The samples of Fig. 1A were used for the analysis. Each dot represents one donor. Young (blue circles, n =30) and elderly (elderly red circles, n=26). ***$p < 0.001$. n.s., not significant.

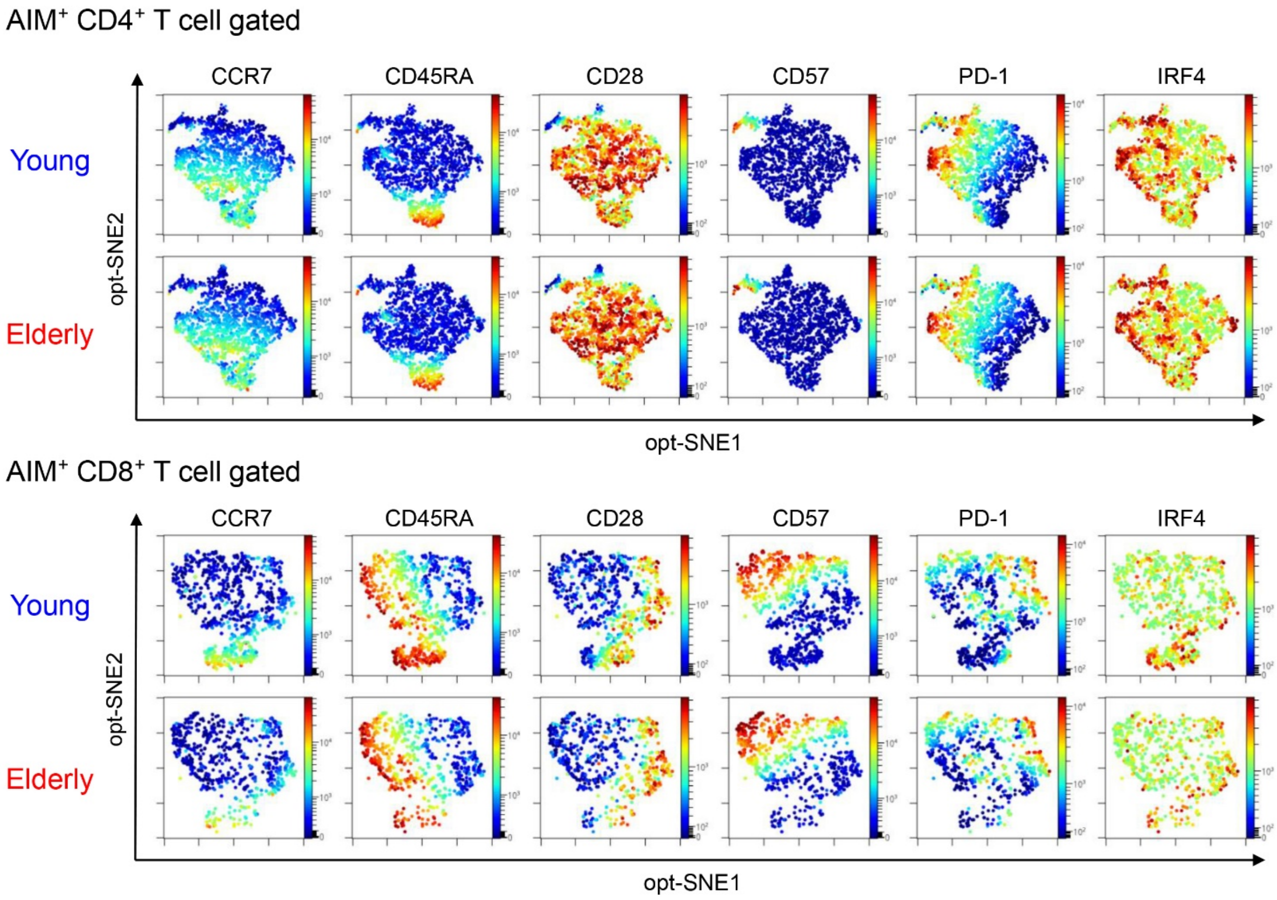

Supplementary Figure 3. opt-SNE mapping of SARS-CoV-2-reactive T cells from the young and elderly cohorts

opt-SNE plots showing the expression of individual markers in the $AIM^{+}CD4^{+}$ (top panels) and $AIM^{+}CD8^{+}$ (bottom panels) T cells in Figure 2 are shown separately for the young (n = 30) and elderly (n = 26) cohorts.

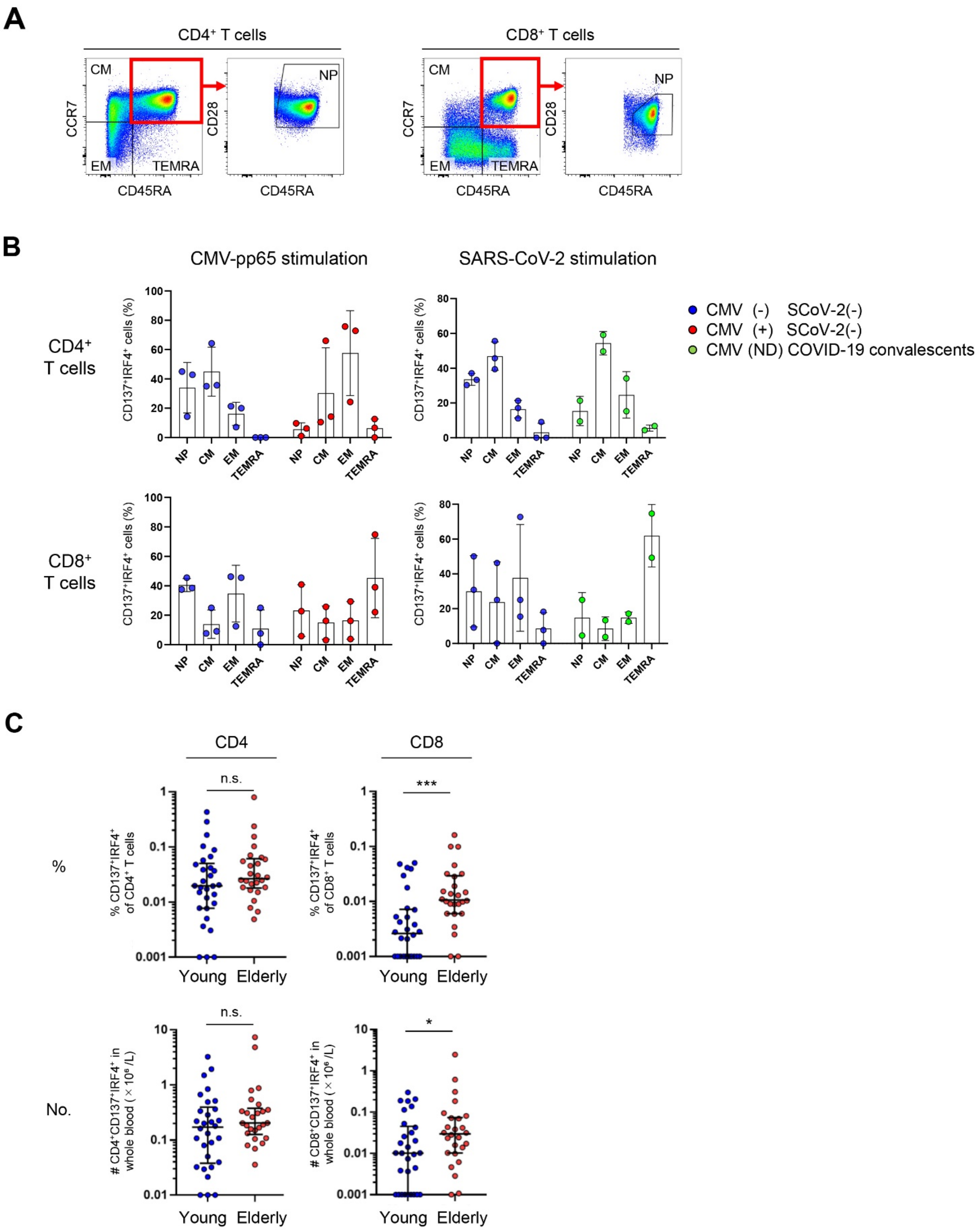
A
CD4+ T cells
CD8+ T cells
CM
EM
TEMRA
NP
CCR7
CD28
CD45RA
B
CMV-pp65 stimulation
SARS-CoV-2 stimulation
CD4+ T cells
CD8+ T cells
CD137+IRF4+ cells (%)
NP CM EM TEMRA
CMV (-) SCoV-2(-)
CMV (+) SCoV-2(-)
CMV (ND) COVID-19 convalescents
C
CD4
CD8
%
No.
% CD137+IRF4+ of CD4+ T cells
% CD137+IRF4+ of CD8+ T cells
# CD4+CD137+IRF4+ in whole blood (×10⁶/L)
# CD8+CD137+IRF4+ in whole blood (×10⁶/L)
n.s.
***
*
Young
Elderly

Supplementary Figure 4. Immunophenotyping of SARS-CoV-2-reactive T cells from young and elderly individuals

(A) Flow cytometry gating strategy for immunophenotypes. The $CD4^+$ and $CD8^+$ T cells defined in Supplementary Fig. 2A were further defined as naïve phenotype (NP; $CD45RA^+CCR7^-CD28^+$), central memory (CM, $CD45RA^-CCR7^+$), effector memory ($CD45RA^-CCR7^-$) and terminally differentiated effector memory cells re-expressing CD45RA (TEMRA, $CD45RA^+CCR7^-$). **(B)** The same samples as in Supplementary Fig. 2B were further analyzed for the immunophenotypes (NP, CM, EM, and TEMRA), as defined in Supplementary Fig. 4A, and the percentages of AIM^+ ($CD137^+IRF4^+$) cells in $CD4^+$ and $CD8^+$ T cells are shown. Data were background subtracted against DW and are shown as the median ± IQR. **(C)** Frequencies (top) and calculated numbers in whole blood (bottom) of AIM^+ ($CD137^+CD154^+$) memory phenotype (CM, EM, and TEMRA) T cells in $CD4^+$ and $CD8^+$ T cells from the young and elderly cohorts. Data were background subtracted against DW and are shown as the median ± IQR. Statistical comparisons across cohorts were performed with the Mann-Whitney test.

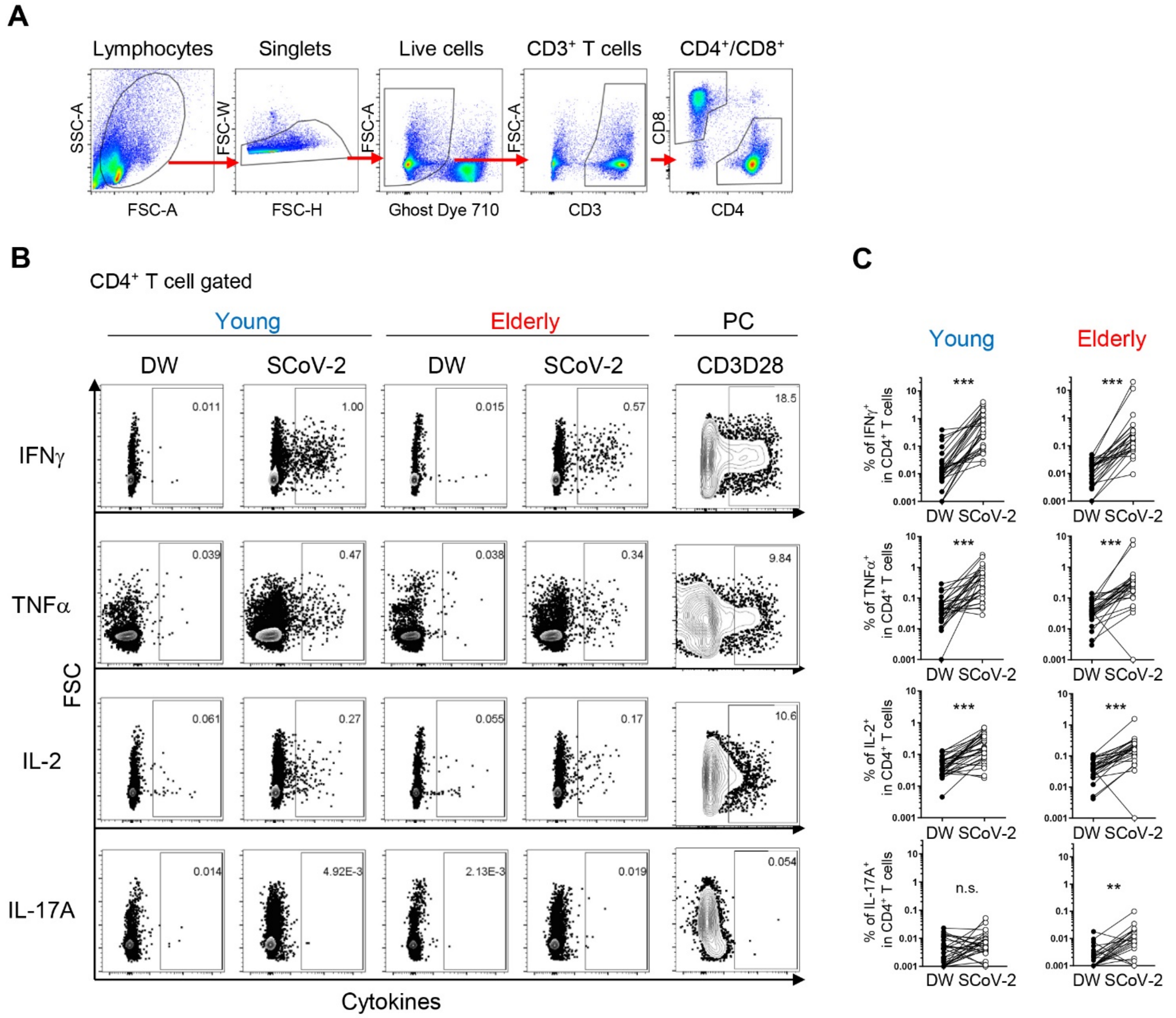

A
Lymphocytes
Singlets
Live cells
CD3+ T cells
CD4+/CD8+
SSC-A
FSC-A
FSC-W
FSC-H
FSC-A
Ghost Dye 710
FSC-A
CD3
CD8
CD4
B
CD4+ T cell gated
Young
Elderly
PC
DW
SCoV-2
DW
SCoV-2
CD3D28
IFNγ
TNFα
IL-2
IL-17A
FSC
Cytokines
0.011
1.00
0.015
0.57
18.5
0.039
0.47
0.038
0.34
9.84
0.061
0.27
0.055
0.17
10.6
0.014
4.92E-3
2.13E-3
0.019
0.054
C
Young
Elderly
% of IFNγ+ in CD4+ T cells
% of TNFα+ in CD4+ T cells
% of IL-2+ in CD4+ T cells
% of IL-17A+ in CD4+ T cells
***
n.s.
**
DW SCoV-2

D

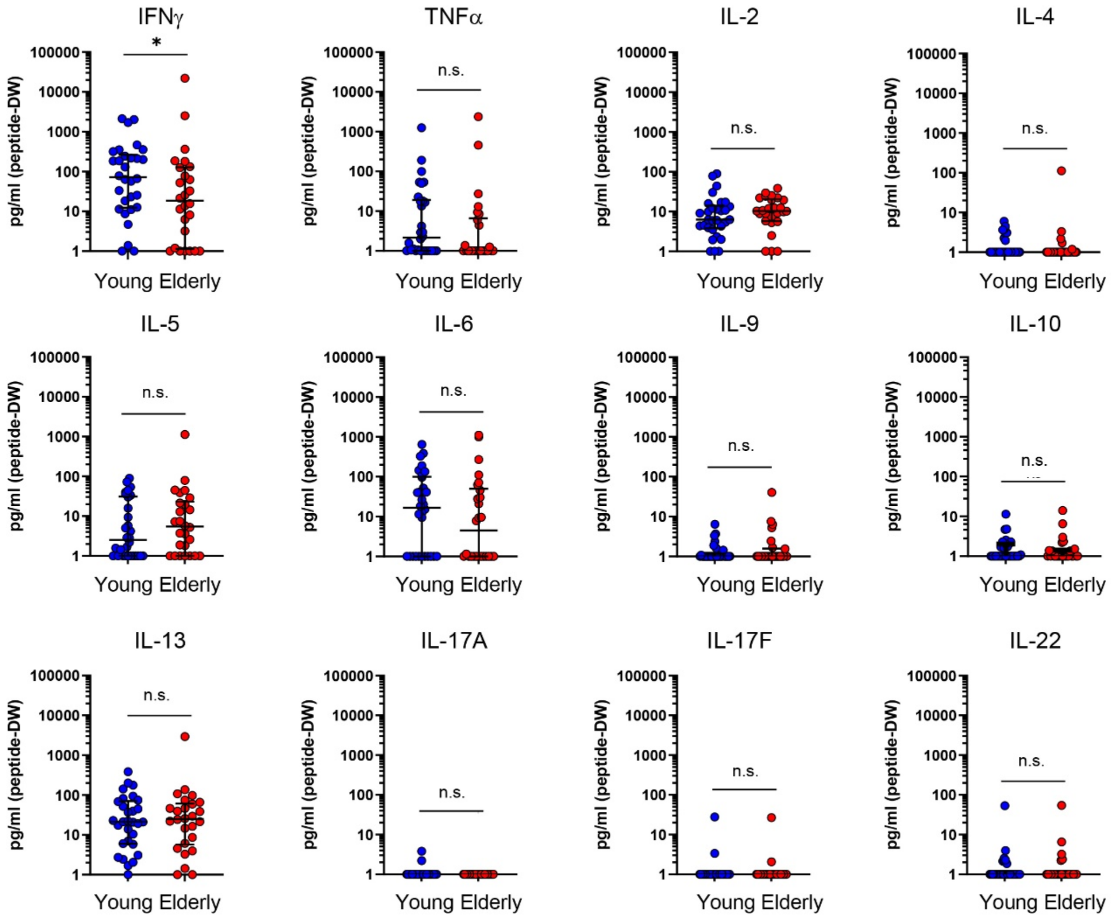
IFNγ
*
TNFα
IL-2
IL-4
IL-5
IL-6
IL-9
IL-10
IL-13
IL-17A
IL-17F
IL-22
n.s.
pg/ml (peptide-DW)
100000
10000
1000
100
10
1
Young Elderly

E

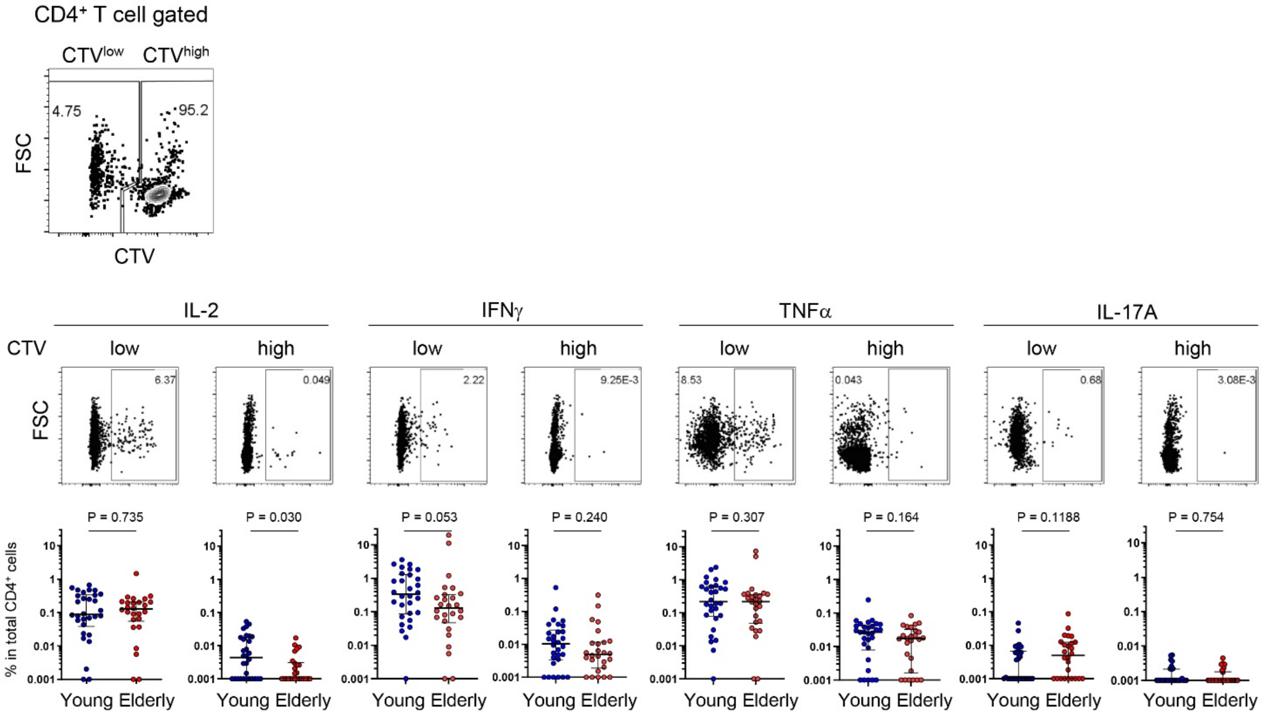
CD4⁺ T cell gated
CTVlow CTVhigh
4.75
95.2
FSC
CTV
IL-2
IFNγ
TNFα
IL-17A
CTV
low
high
6.37
0.049
2.22
9.25E-3
8.53
0.043
0.68
3.08E-3
P = 0.735
P = 0.030
P = 0.053
P = 0.240
P = 0.307
P = 0.164
P = 0.1188
P = 0.754
% in total CD4⁺ cells
10
1
0.1
0.01
0.001
Young Elderly

Supplementary Figure 5. SARS-CoV-2-specific cytokine production in young and elderly $CD4^+$ T cells

(A) Representative gating strategy to detect $CD3^+$ T cells, $CD4^+$ T cells and $CD8^+$ T cells for intracellular cytokine staining. Briefly, lymphocytes were gated out of all events, and doublet exclusion was performed. Live cells were gated as Ghost Dye™ Red 710^-. T cells were then gated as $CD3^+$ and further subdivided into $CD4^+$ and $CD8^+$ populations. $CD4^+$ and $CD8^+$ T cells were further analyzed with antibodies against various cytokines. **(B)** Representative flow cytometry plots showing the expression of IFNγ, TNFα, IL-2, and IL-17A in the total $CD4^+$ T cell populations of young ($n = 30$) and elderly ($n = 25$) subjects after 6 days of stimulation with SARS-CoV-2 peptide pool (SCoV-2) or negative control (DW), as detected by the intracellular staining of each cytokine. Most right panels show positive controls (PC); PBMCs from the young were stimulated with anti-CD3/CD28 Dynabeads. One sample from the elderly cohort was excluded from the analysis due to the low number of PBMCs obtained. **(C)** Percentages of $IFN\gamma^+$, $TNF\alpha^+$, $IL\text{-}2^+$, and $IL\text{-}17A^+$ cells in $CD4^+$ T cells are shown between DW (DW) and SARS-CoV-2 peptide pool (SCoV-2) stimulation. Each dot represents one donor. Pairwise comparisons were performed with the Wilcoxon test. $^*p < 0.05$, $^{**}p < 0.01$, $^{***}p < 0.001$. **(D)** Cytokines in the supernatants of AIM assays 3 days after stimulation with the SARS-CoV-2 peptide pool (SCoV-2) or negative control (DW) were detected using bead-based cytokine assays. Data were background subtracted against DW and are shown as the median ± IQR. Samples were from young ($n = 30$) and elderly donors ($n = 25$). Each dot represents one donor. Statistical comparisons across cohorts were performed with the Mann-Whitney test. $^*p < 0.05$, n.s., not significant. **(E)** Representative FCM plots showing CTV and FSC gated on total $CD4^+$ T cells 6 days after stimulation with SARS-CoV-2 peptides. Boxed gates define CTV^{low} and CTV^{high} cells (top). Representative FCM plots showing the expression of IL-2, IFNγ, TNFα, and IL-17A among CTV^{low} and CTV^{high}cells. Numbers indicate percentages in the drawn gates (middle). Percentages of $IL\text{-}2^+$, $IFN\gamma^+$, $TNF\alpha^+$, and $IL\text{-}17A^+$ cells in total $CD4^+$ T cells from the young (blue circles) and elderly (red circles) cohorts. Data were background subtracted against DW and are shown as the median ± IQR. Each dot represents one donor. Statistical comparisons across cohorts were performed with the Mann-Whitney test (bottom).

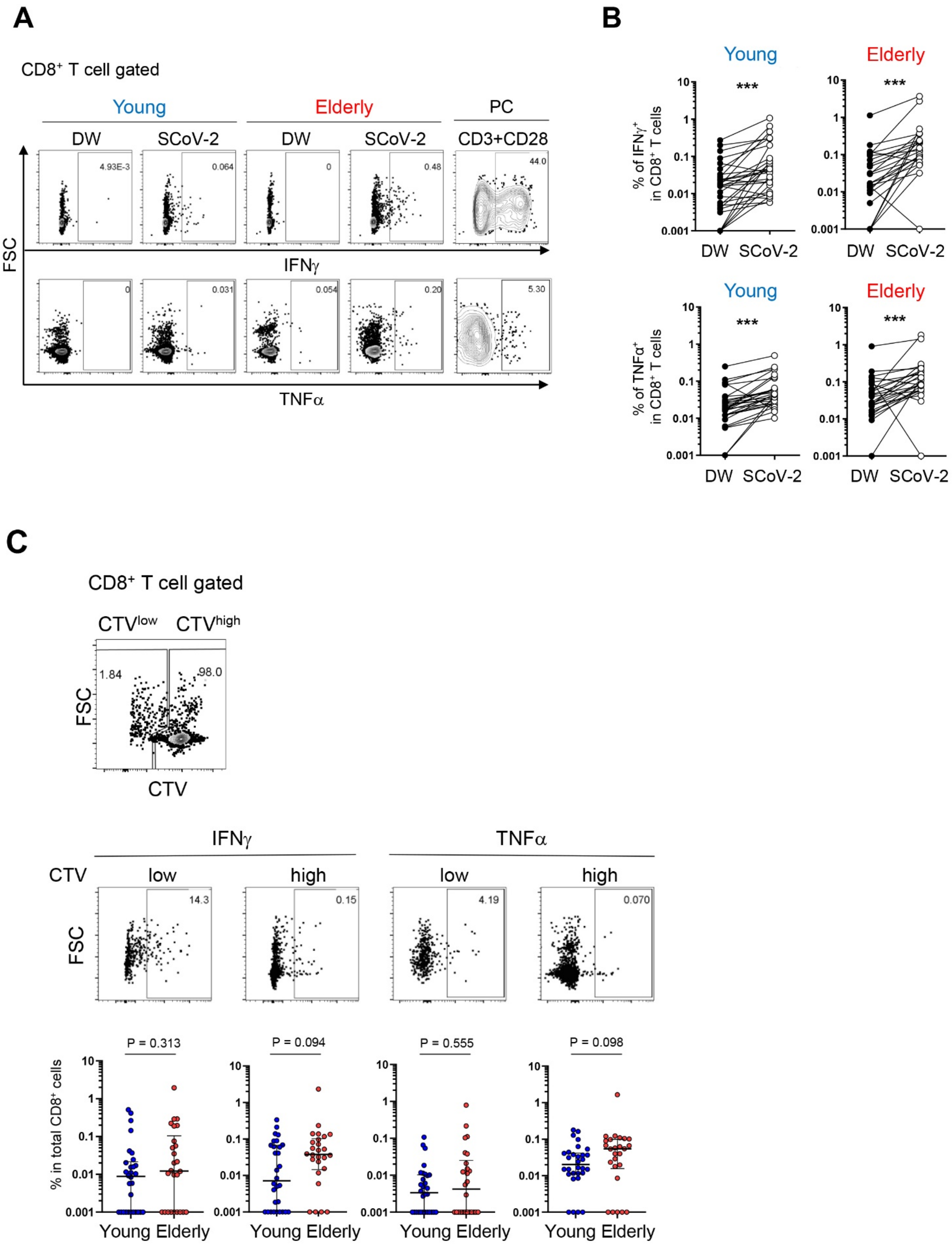
A
CD8⁺ T cell gated
Young
Elderly
PC
DW
SCoV-2
DW
SCoV-2
CD3+CD28
4.93E-3
0.064
0
0.48
44.0
FSC
IFNγ
0
0.031
0.054
0.20
5.30
TNFα
B
Young
Elderly
% of IFNγ⁺ in CD8⁺ T cells
***
***
DW SCoV-2
DW SCoV-2
Young
Elderly
% of TNFα⁺ in CD8⁺ T cells
***
***
DW SCoV-2
DW SCoV-2
C
CD8⁺ T cell gated
CTV^low CTV^high
1.84
98.0
FSC
CTV
IFNγ
TNFα
CTV
low
high
low
high
14.3
0.15
4.19
0.070
FSC
P = 0.313
P = 0.094
P = 0.555
P = 0.098
% in total CD8⁺ cells
Young Elderly
Young Elderly
Young Elderly
Young Elderly

Supplementary Figure 6. SARS-CoV-2-specific cytokine production from young and elderly $CD8^+$ T cells

(A) Representative flow cytometry plots showing the expressions of IFNγ and TNFα among total $CD8^+$ T cells from young (n = 30) and elderly (n = 25) subjects 6 days after stimulation with the SARS-CoV-2 peptide pool (SCoV-2) or negative control (DW). Each cytokine was detected by intracellular staining. Most right panels show positive controls (PC); PBMCs from the young were stimulated with anti-CD3/CD28 dynabeads. One sample from the elderly cohort was excluded from the analysis due to the low number of PBMCs obtained. Each dot represents one donor. **(B)** Percentages of $IFN\gamma^+$ (top panel) and $TNF\alpha^+$ (bottom panel) cells in $CD8^+$ T cells between DW- and SARS-CoV-2 peptide pool (SCoV-2)- stimulated cells. Each dot represents one donor. Pairwise comparisons were performed with the Wilcoxon test. ***$p < 0.001$. **(C)** Representative FCM plots showing CTV and FSC gated on total $CD8^+$ T cells 6 days after stimulation with SARS-CoV-2 peptides. Boxed gates define CTV^{low} and CTV^{high} cells (top). Representative FCM plots showing the expression of IFNγ and TNFα among CTV^{low} and CTV^{high}cells. Numbers indicate percentages in the drawn gates (middle). Percentages of $IFN\gamma^+$ and $TNF\alpha^+$ cells in total $CD8^+$ T cells from the young (blue circles) and elderly (red circles) cohorts. Data were background subtracted against DW and are shown as the median ± IQR. Each dot represents one donor. Statistical comparisons across cohorts were performed with the Mann-Whitney test (bottom).

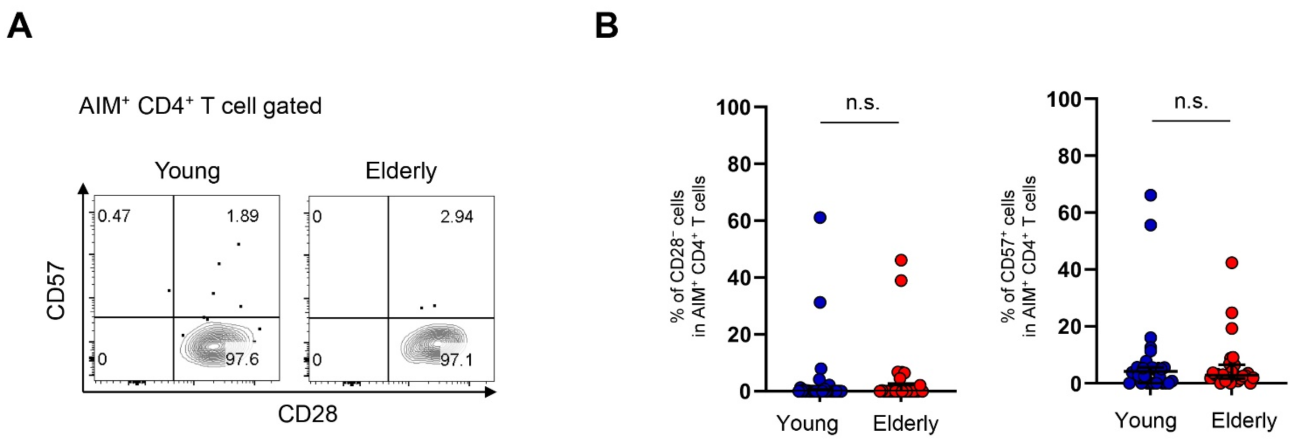

Supplementary Figure 7. CD28 and CD57 expression in SARS-CoV-2-reactive CD4$^+$ T cells

(A) Representative flow cytometry plots showing the expression of CD57 and CD28 among CD4$^+$AIM$^+$ T cells after stimulation with the SARS-CoV-2 peptide pool in the AIM assay. Numbers indicate percentages in the gates. **(B)** Frequency of CD28$^-$ and CD57$^+$ cells among SARS-CoV-2-specific AIM$^+$ CD4$^+$ T cells in young (n = 30) and elderly (n = 25) cohorts. Each dot represents one donor. Data are shown as the median ± IQR. Statistical comparisons across cohorts were performed using the Mann-Whitney test. n.s., not significant.

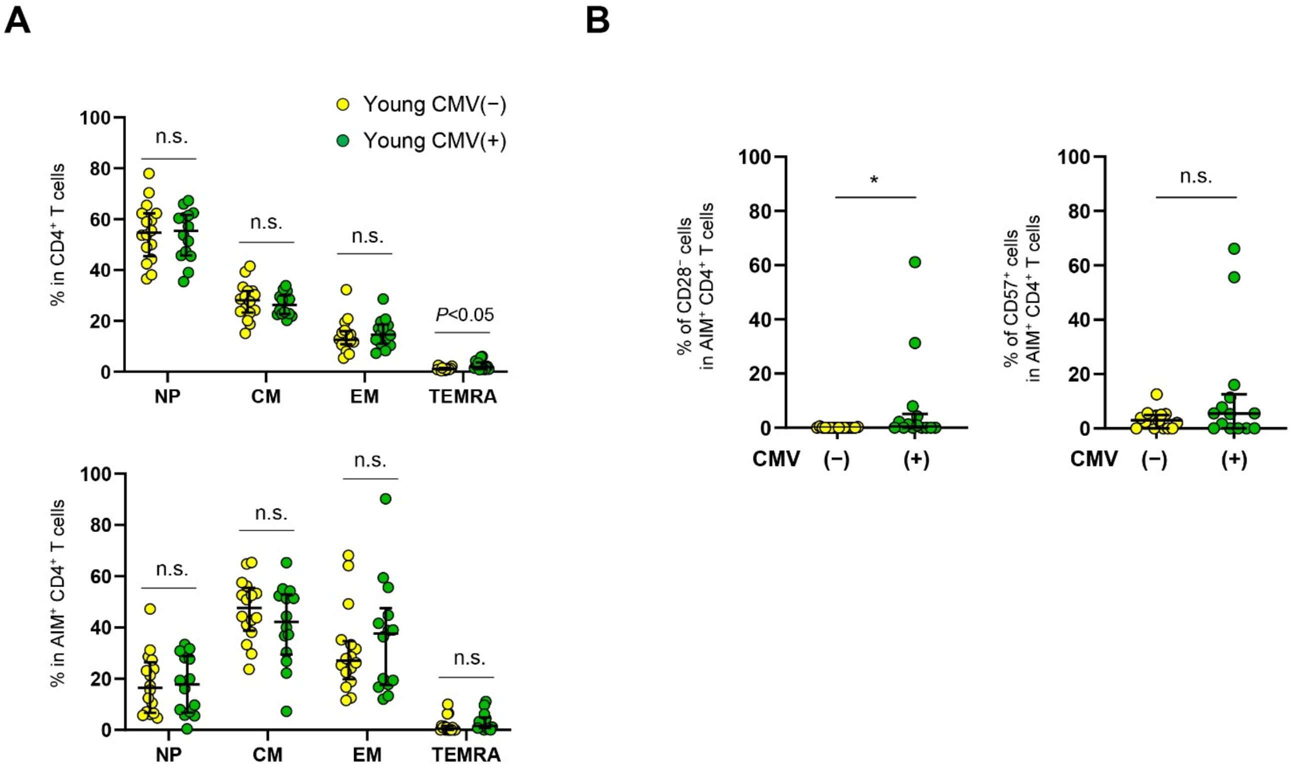

Supplementary Figure 8. Immunophenotyping of SARS-CoV-2-reactive CD4$^+$ T cells from the young CMV$^+$ and CMV$^-$ individuals

(A) Frequency of NP, CM, EM, and TEMRA cells in total CD4 $^+$ (top) and SARS-CoV-2-specific AIM$^+$ CD4$^+$ T cells (bottom) of CMV$^+$ (n = 14) and CMV$^-$ (n = 16) individuals. **(B)** Frequency of CD28$^-$ and CD57$^+$ cells among SARS-CoV-2-specific AIM$^+$CD4$^+$ T cells in CMV$^+$ and CMV$^-$ individuals. Each dot represents one donor. Data are shown as the median ± IQR. Statistical comparisons across cohorts were performed using the Mann-Whitney test. n.s., not significant.

Marker	Clone	Fluorochrome	Manufacturer
CD4	SK3	BUV395	BD Bioscience
CD45RA	HI100	BV421	SONY
CD8a	RPA-T8	BV510	BioLegend
CD3	UCHT1	BV605	BioLegend
CD57	HCD57	FITC	BioLegend
IRF4	IRF4.3F4	PE	BioLegend
PD-1	EH12.2H7	PE/Dazzle	SONY
CD28	CD28.2	PerCP-Cy5.5	BioLegend
CD154	24-31	PE/Cy7	BioLegend
CD137	4B1-1	APC	BioLegend
Viability Dye	N/A	Ghost Dye Red 710	TONBO
CCR7	G043H7	APC/Cy7	BioLegend

Supplementary Table 1. Flow cytometry panel

Marker	Clone	Fluorochrome	Manufacturer
CD4	SK3	BUV395	BD Bioscience
Cell proliferation	N/A	CellTrace Violet	Thermo Fisher Scientific
CD8a	RPA-T8	FITC	TONBO
CD3	UCHT1	GrnPE	BioLegend
TNFα	MAb11	PE/Dazzle	BioLegend
IL-2	MQ1-17H12	PE/Cy7	BioLegend
IFNγ	4S.B3	APC	BioLegend
Viability Dye	N/A	Ghost Dye Red 710	TONBO
IL-17A	BL168	APC/Cy7	BioLegend

Supplementary Table 2. Intracellular staining panel
